# Placebo and Non-specific Effects in Reconsolidation-Based Treatment for Arachnophobia

**DOI:** 10.3389/fpsyt.2021.775770

**Published:** 2021-11-12

**Authors:** James W. B. Elsey, Merel Kindt

**Affiliations:** Department of Clinical Psychology, University of Amsterdam, Amsterdam, Netherlands

**Keywords:** memory reconsolidation, propranolol, placebo, arachnophobia, fear and anxiety, spider, clinical translation

## Abstract

The idea that maladaptive memories may be rendered susceptible to interference after reactivation raises the possibility of reactivating and neutralizing clinically-relevant emotional memories. In this study, we sought to investigate the feasibility of such a “reconsolidation-based” intervention for arachnophobia, drawing upon previous research that successfully reduced fear of spiders in a subclinical sample. In Experiment 1, we piloted several reactivation procedures for conducting a reconsolidation-based treatment for arachnophobic individuals. All procedures involved some form of brief exposure to a fear-provoking spider, followed by the administration of 40 mg propranolol. In Experiment 2, we conducted a double-blind, placebo-controlled assessment of one procedure tested in Experiment 1. In Experiment 1, we found that most reactivation procedures produced drops in self-reported fear of spiders from pre- to post-treatment, including fear declines that were apparent up to 6- and even 14-months later. However, in Experiment 2, we found no evidence that the participants receiving propranolol were better off than those who received placebo. While our findings are limited by the small sample sizes used, they nevertheless show a different pattern of responses than was observed in a previous reconsolidation-based intervention for *subclinical* spider fearful participants. Alterations to the protocol made to accommodate the clinical participants may have led to greater opportunities for non-specific effects (e.g., exposure, placebo effects) to drive change in the participants. Our findings highlight both the challenges of translating reconsolidation-based procedures into clinical interventions, as well as the importance of controls for non-specific effects in reconsolidation-based research.

## Introduction

Cognitive behavioral therapies (CBT) are currently the most empirically supported method of treatment for anxiety disorders (1). Nevertheless, many patients undergoing CBT fail to benefit, and of those who do show an initial response, many relapse (2–4). Influential models of the principal components of CBT—cognitive therapy (5) and exposure (6, 7)—may help to explain relapse. These models suggest that, rather than directly altering maladaptive cognitions, learned behaviors, and affective responses, cognitive behavioral interventions generate alternative adaptive representations in memory that compete with maladaptive ones for control over behavior. Because maladaptive memory traces remain intact even after successful therapy, they can resurface with the passage of time, in novel situations, or when the patient is highly stressed, leading to relapse.

While the assumption that emotional memory may be indelible guided translational research for some time (8), more recent insights from neuroscience challenge this view (9). It is now hypothesized that, under certain conditions, reactivation can render a memory transiently labile, requiring a process of restabilization in order to persist. Findings consistent with this retrieval-dependent malleability of memory, known as reconsolidation, have been found across a host of animal models and in human experiments, although not conclusively established (10). Crucially, because restabilization is thought to rely upon *de novo* protein synthesis [though see (11)], drugs that interfere with this process can produce amnesia. When administered so as to disrupt the putative process of reconsolidation, the noradrenergic betablocker propranolol has demonstrated efficacy in reducing conditioned responding to threat stimuli in both animals and humans (12, 13). In human Pavlovian fear conditioning experiments, participants receiving propranolol timed to interfere with reconsolidation have been found to display attenuation of conditioned fear responses, with less spontaneous recovery, less renewal, and fewer savings of the original memory trace compared with those receiving placebo and extinction training (13–15).

An exciting prospect of such research is that, by disrupting the restabilization of maladaptive emotional memories in clinical disorders, it may be possible to directly attenuate or even effectively neutralize them. Such an approach could provide a rapid and long-lasting treatment for anxiety disorders, in which maladaptive emotional memories are thought to play a key role (16). However, such translation is by no means simple: though they provide an informative model, fear memories induced in the lab are far-removed from clinically significant anxiety and fear, which is stronger, more enduring, and often accompanied by other comorbid symptoms.

Attempts have been made to translate such an approach to the treatment of post-traumatic stress disorder, with both promising and disappointing results (17–19). A more recent study also aimed to tackle a naturalistic fear of public speaking using a reconsolidation-based approach, but found that propranolol + reactivation did not outperform placebo + reactivation [(20); throughout, we use the term “reconsolidation-based” to refer to the ideas underpinning such treatments, but not as a conclusive statement that reconsolidation necessarily underpins any observed effects]. Perhaps the most convincing demonstration of the prospect of harnessing reconsolidation to tackle naturalistic fears was a placebo-controlled study of participants with a subclinical fear of spiders (21). In Soeter and Kindts' study, participants were briefly exposed to a tarantula to reactivate their fear, after which they received 40 mg oral propranolol or a placebo. A third group received propranolol without fear reactivation, to control for a pure drug effect. Propranolol combined with reactivation was found to produce an abrupt change in approach behavior and fear responses toward spiders at follow-up testing, with participants able to touch or even hold spiders, as well as experiencing less subjective fear than at treatment and pre-test. Large reductions in scores on the Spider Phobia Questionnaire (SPQ) (22) were also observed after 3 months, and these behavioral and self-reported changes in spider fear were maintained at a 1-year follow-up. Despite repeated exposure to spiders at several follow-up tests, the two control groups had no significant changes in fear of spiders.

These findings highlight that specific subclinical fears, and possibly by extension clinical phobias, could be amenable to an intervention based on the principles of reconsolidation. Translating experimental research on reconsolidation to specific phobias may prove informative in further testing whether reconsolidation-based approaches are a viable method for tackling strong and long-lasting maladaptive memories. Not only are specific phobias valuable targets in their own right, but positive findings for a clinically significant phobia could also provide a stronger case for the investigation of reconsolidation-based interventions in arguably more complex anxiety disorders, such as panic and social anxiety disorder. However, the optimal means of performing reconsolidation-based treatments for clinically significant fears remain unknown, in terms of both efficacy and practical feasibility. Practically, clinically phobic patients might find the types of reactivation used in subclinical populations too confronting or difficult to undertake. In addition, factors such as the age and strength of targeted memories, which more basic science research has shown to be important in whether or not memory reconsolidation can be induced or disrupted (23), may reduce the efficacy of reconsolidation-based procedures in clinical populations.

In the present experiments, we sought to exploratorily pilot several possible means of reactivating the fears of arachnophobic patients. These patients differed from those who participated in Soeter and Kindt (21) in that they were required to meet full criteria for a specific phobia, whereas those in Soeter and Kindt (21) did not need to experience interference in their lives due to their phobia. These patients were recruited on the basis of either contacting a clinical center at the University of Amsterdam that offers reconsolidation-based interventions, or through responding to an advertisement offering participation in a trial of such treatments. Participants in Soeter and Kindt (21) were not specifically seeking to tackle their fears.

In Experiment 1, we tested whether the same procedure as that used in Soeter and Kindt (21) could simply be used directly in clinical patients. Of particular importance, the reactivation procedure in Soeter and Kindt entailed an element of deception, as participants were led to believe they would have to touch the spider in the treatment session, when in fact they would not. This type of reactivation procedure might lack clinical utility, not only because some patients may immediately balk at the prospect of having to touch a tarantula, but also because knowledge about the intervention is likely to spread with its uptake. For example, media coverage or patient reports describing exactly what happens during such a treatment might mean that future participants would already know they do not have to touch the tarantula. If this deception is essential to the treatment effect, it might be undermined once people know what to expect. Based upon informal feedback from patients and clinical observations, we further piloted several adjusted reactivation procedures that did not involve deception, to assess whether the practicalities of the reactivation were more feasible, and if any procedure clearly outperformed the others. Because the development of these procedures was made on an ongoing basis, we did not have specific expectations that one type of reactivation would strictly come out as better than others. As will be seen in the results, each procedure on average produced reductions in phobic responding. However, we expected that many of the changes observed in patients might be achieved through exposure, placebo, or other non-specific effects. Hence, in Experiment 2, we performed a double-blind, placebo-controlled assessment of the standard reactivation procedure. If the observed reductions in fear of spiders in Experiment 1 were attributable to post-reactivation propranolol disrupting fear memory reconsolidation, then it would be expected that reductions in the propranolol group would be greater than in the placebo group. This would be expressed as greater reductions in distress from pre- to post-treatment, and greater reductions in SPQ scores over time. Higher performance on the post-treatment behavioral approach test (BAT) would also be indicative of such an effect, though not conclusive, owing to the absence of a pre-treatment BAT.

## Methods

### Ethical Approval

All procedures were approved by the University of Amsterdam ethical review board (2017-CP-7625), the research was performed in accordance with relevant guidelines/regulations, and participants gave informed consent.

### Experiment 1

#### Recruitment and Exclusion Criteria

Participants were recruited through referrals to a clinical facility at the university and with the use of Facebook advertisements. Participants were required to be aged 17 or above, and to have received approval from their doctor to take propranolol. Participants additionally underwent a medical screening to assess any contraindications for receiving propranolol (full criteria in Supplementary Material section: Medical Inclusion/Exclusion Criteria for Propranolol Administration), had not been diagnosed with other mental health conditions, and were not taking psychoactive medication. Participants additionally underwent a Structured Clinical Interview for DSM-5 specific phobia diagnosis, and were required to meet the criteria for specific phobia.

Participants were excluded if they indicated they were unlikely to be afraid of a tarantula, for example if they were almost exclusively afraid of “harvestman” spiders. Participants were also excluded if they did not properly follow instructions in the treatment session, or if they were judged to have been abnormally unafraid of the tarantula (for a phobic person). Such exclusion decisions were made before the follow-up session, as seeing outcomes might have influenced decisions of inclusion. We do not have information on the full number of people who were screened out based on an initial online screener. An exclusion chart is presented in the Supplementary Materials. In Experiment 1, 58 participants came to intake, and 43 completed all sessions and were included in analyses. Of the 15 excluded, reasons were: not responding to contact/dropping out before completing all sessions (*n* = 7), fear almost exclusively of a different type of spider (*n* = 2), low heart rate (*n* = 3), not following the procedures sufficiently in session 2 (*n* = 2), and appearing unafraid of the tarantula (*n* = 1).

Table 1 shows sample sizes and descriptive statistics for each group in Experiment 1. The questionnaires used are described in the Materials section, and statistical results are presented in the Results section Baseline characteristics.

**Table 1:** Baseline characteristics of groups in Experiment 1.

		**Mean**	**SD**	**Min**	**Max**	***BF_***Condition***_***	***p* condition**
Age	Standard	36.67	9.53	22	54	0.455	0.248
	Enclosure	29.45	10.62	18	49		
	Loose tarantula	31.25	8.77	17	43		
	Observe	35.00	7.25	27	47		
SPQ	Standard	24.50	3.92	18	30	0.365	0.308
	Enclosure	23.00	1.90	19	25		
	Loose tarantula	24.42	2.75	19	28		
	Observe	22.25	3.58	16	27		
ASI	Standard	6.75	3.31	2	12	1.906	0.04
	Enclosure	13.45	6.50	6	25		
	Loose tarantula	13.42	9.98	1	40		
	Observe	8.00	4.47	4	15		
PHQ	Standard	4.08	2.78	0	11	0.3	0.389
	Enclosure	3.27	2.69	1	11		
	Loose tarantula	4.33	3.63	1	14		
	Observe	2.25	1.39	1	5		
STAIT	Standard	38.50	9.31	26	55	0.577	0.166
	Enclosure	34.64	6.70	24	45		
	Loose tarantula	36.13	6.09	27	48		
	Observe	31.06	5.54	20	36		
						**Contingency:**	
		**M:F**	***n***			***Bayes factor***	***p*** **-value**
Sex	Standard	0:12	12			0.784	0.608
	Enclosure	1:10	11				
	Loose tarantula	1:11	12				
	Observe	0:8	8				

*BF_Condition_, Bayes factor for inclusion of condition in ANOVA; p condition, p-value for difference between groups in ANOVA*.

### Materials and Measures

#### Propranolol

Propranolol (40 mg) was administered orally as a pill, within 5 min after confronting a tarantula/spider in the treatment session (see Procedures below). Propranolol reaches peak bioavailability between 1 and 2 h after ingestion and has been found to effectively neutralize fear memory even when given 1 h after reactivation, indicating that timing the pill administration for shortly after reactivation fits well within the “reconsolidation window” (24). Giving propranolol after reactivation also avoids the possibility that pill administration affects something occurring during reactivation itself, allowing for clearer inferences if the administration is found to be effective. Propranolol pills were made by Accord Healthcare Ltd. (UK), and provided along with placebo pills by Huygens Apothecary (NL). A 40 mg dose was used in Soeter and Kindts' (21) successful intervention for subclinical spider fear, and in successful experimental reconsolidation-based studies, with effects irrespective of participant body mass (25). Each pill was placed in an envelope shortly before the treatment session and handed to the participant single-blind.

#### Tarantula Behavioral Approach Test

Participants performed a BAT to assess the level of interaction with a spider that they were capable of in the post-treatment session. An adult *grammostola porteri* tarantula was placed in a 30 cm^3^ glass terrarium. Participants were asked to approach a line on the floor placed 30 cm from the table on which the terrarium sat, at which point the door was opened and participants were asked to touch the spider. A time limit of 7.5 min was placed on the BAT, which could also be terminated by the participant upon request. Participants also reported how much distress/tension they felt whilst standing at the line, before trying to touch the tarantula. Scoring for the test was: 0 = unable to reach the line, 1 = standing at least at the line, 2 = touching the terrarium, 3 = putting hand in the terrarium, 4 = touching the tarantula.

#### Standardized Questionnaires

Multiple psychometrically-validated questionnaires were used to assess participants' level of fear of spiders [SPQ: (22); Fear of Spiders Questionnaire (FSQ): (26)], their state and trait anxiety [State-Trait Anxiety Index (STAI): (27)], possible depressive symptoms [Patient Health Questionnaire (PHQ): (28)], and sensitivity to anxiety-relevant sensations [Anxiety Sensitivity Index (ASI): (29)], and a Subjective Units of Distress Scale [SUDS: (30, 31)]. Full details on these questionnaires are presented in Supplementary Material section Questionnaire Information.

Participants also responded to several eight-point Likert scale items that have been used in previous studies, in which participants indicated how much fear they have of spiders, how much avoidance they engage in, how much their fear interferes with their lives, as well as how much they trust in typical treatments for phobias, and in the experimental treatment. These unvalidated measures were not used for analyses, but are provided in the open access data.

### Procedure

**Figure 1:**
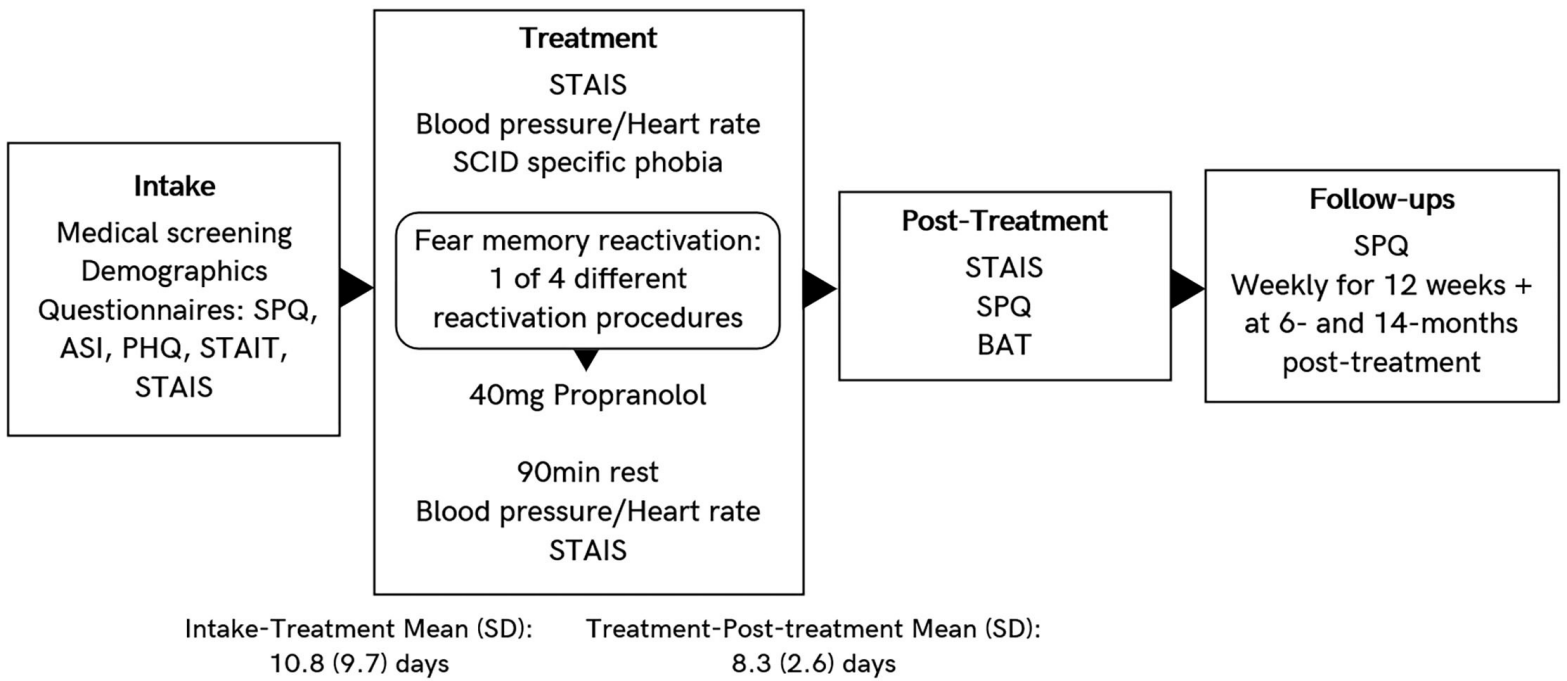
Schematic overview of Experiment 1 procedure. Different reactivation procedures are “standard,” “observe,” “loose tarantula,” and “enclosure,” as described in methods section.

Figure 1 provides a schematic overview of the procedure for Experiment 1, and when different measures were obtained.

#### Intake Session

Participants read an information brochure, and the researcher answered any questions the participant might have. Notably, the information indicated that there was a chance of receiving placebo, although in fact all participants received propranolol in Experiment 1. Participants gave their informed consent to the procedure and then underwent a medical screening. The session was terminated if participants did not pass the medical screening. Participants then completed the STAI, PHQ, ASI, and SPQ. The researcher organized a date for the treatment and follow-up sessions and concluded the intake.

#### Treatment Session

Treatment sessions took place on average 11 days after the intake session. Due to scheduling conflicts, one treatment session took place 52 days after intake. Treatment sessions of all other participants took place 25 days or less since intake. In all treatment sessions, the researcher from Session 1 introduced the participant to the clinician who then led the session. The duration of exposure for each condition is presented in Supplementary Material section Duration of Exposure at Treatment, with analyses suggesting no robust differences between groups but a slight tendency for the enclosure condition to last longer than the others. Participants in different conditions underwent one of four different types of fear memory reactivation detailed below (*Standard, Observe, Enclosure*, or *Loose Tarantula*). The “Standard” procedure was named as such because it was based on the reactivation session used as standard in previous research with subclinical participants (21).

For all treatment sessions, participants began the session by filling in the STAI-State and then had their heart rate and blood pressure checked. The clinician then interviewed the participant about their fear, including administration of the SCID-5 for specific phobia. The clinician then explained the rationale for the reconsolidation-based treatment, noting the need for some kind of fear memory reactivation, followed by propranolol. Participants then underwent one of the reactivation procedures below. Assignment was not random but sequential, with batches of participants undergoing one procedure, as each procedure was devised based upon observation of participants and informal interviews with them at follow-up.

#### Standard

After being given the rationale for the treatment, participants were informed that they would be required to approach and touch a live tarantula. The participant was then led to another room, in which a tarantula was held in a glass terrarium, on top of a table. The participants were encouraged to approach the tarantula and to stand at a line placed 30 cm from the table. The clinician stood close to the participant and to the terrarium, and encouraged the participant to stay still and observe the tarantula, whilst opening the front door of the terrarium. In order to ensure the participant understood that the spider was real, the clinician used a pipette to gently spray the tarantula's back with water, which usually causes it to walk around. The clinician asked the participant what they would be most afraid of happening when they touch the tarantula, how likely this would be to occur (0–100), what they would feel when they touch the tarantula, and how strong this would be (0–100). Finally, the clinician told the patient they were about to touch the tarantula, and asked how much tension/distress they currently felt (0–100). After the participant answered this question, they were told that they could in fact return to the other room, and did not need to touch the tarantula. Twelve patients were assigned to this reactivation procedure.

#### Observe

The “Observe” condition was the same in all respects as the standard session, with the exception that participants were not told that they would have to touch the tarantula, but instead to approach to within 30 cm of the terrarium and to observe the spider as it moved, without themselves moving backwards. This variation was performed because the use of deception regarding the touching of the tarantula in the standard procedure could be undermined if the procedure becomes more widely used. Eleven patients were assigned to this reactivation procedure.

#### Enclosure

The participant was led to a different room, in which there was a 2-m × 2-m enclosure, with a smaller but faster moving tarantula in the center (*Hapalopus Sp. Columbia*, approximately 5 cm legspan). The participant was required to enter the enclosure in their bare feet, and use a paint brush to lightly touch the tarantula to make it move. The participant was encouraged not to run away when touching the tarantula, but to stay still and observe what happens. The participant was asked the same questions as in the standard condition, touched the tarantula with the brush two times, and then exited the enclosure. This condition was used because some participants noted the obvious difference between a very large tarantula and the normal sort of spiders they encounter; the dwarf tarantula looks more like a large, typical spider, and moves more quickly than a full-sized tarantula. Twelve patients were assigned to this reactivation procedure.

#### Loose Tarantula

The loose tarantula condition was the same as the Observe condition, with the exception that the tarantula was not held in the terrarium, but rather placed loose on the table. If the tarantula appeared to get too close to the edge of the table, the researcher or clinician would use a clipboard as a barrier to prevent it from going off the edge. The loose tarantula condition was used because informal conversation in follow-up sessions with participants suggested that they felt the terrarium provided a very high degree of control that was not like in their usual spider experiences. Certain possibilities, for example that the spider could easily jump on them, were not so concerning as usual. Eight patients were assigned to this reactivation procedure.

#### End of Treatment Session

After all reactivation types, participants returned to an adjoining room with no spider in it, and the clinician praised them for facing their fear. The participant then received the propranolol pill, and was led to a waiting room by the researcher from the first session. The room included a comfortable chair and light reading material. Participants were informed that they could read the provided magazines or their own reading material, but should not use a laptop or mobile phone during the waiting period. The participant was left alone in the room, with the researcher briefly checking on the participant every 30 min, until 90 min after taking the pill. At this point, the participant filled out the STAI-State again, and had their blood pressure and heart rate checked a second time, and the session was concluded.

#### Post-treatment Session

Post-treatment sessions took place on average 8 days after the treatment session, and all post-treatment sessions took place at or <15 days post-treatment. At the beginning of the post-treatment session, the participant again filled in the STAI-State and SPQ. Now, all participants were informed that they would be asked to touch the tarantula. Notably, participants in the Enclosure condition were informed that this would be a different tarantula to the previous session. Participants then approached the tarantula using the same procedure and questions as in the Standard treatment procedure, but in this instance, once they were asked to touch the tarantula, they were actually allowed to do so—resulting in the Tarantula BAT score. The BAT scoring was concluded if the participant touched the tarantula, told the researcher that they could not go any further, or if the BAT time reached 7.5 min. After finishing the BAT, the experimenter offered to demonstrate touching the tarantula, and gave the participant another opportunity to stay a little longer observing or interacting with the tarantula if they wished.

For the participants in the Standard, Observe, and Loose Tarantula conditions, the researcher then led the participant back to the starting room and asked them if they had any thoughts or feelings about what they had done, and the treatment experience. Participants in the Enclosure condition then went to the enclosure and conducted the same task they had done in their treatment session, followed by being asked about their thoughts and feelings regarding the procedure. After finishing all assessments, participants were informed that they had received propranolol in the treatment session. Participants were asked to confirm that they would complete the weekly questionnaire follow-ups, and also asked to consider investigating some places they might previously have been afraid of encountering spiders to see how they now felt.

#### Questionnaire Follow-Up

Participants were followed up with weekly SPQ questionnaires for a 12-week period, starting from the week after the post-treatment session (due to a technical error, some participants were sent a total of 13 weekly follow-up assessments). We also attempted to reach participants 6 and 14 months after the treatment session with the SPQ.

### Analytic Approach

Our key outcome variables in both experiments were BAT results, distress scores during the treatment session vs. post-treatment BAT, and SPQ scores over time. STAI-State scores were collected to maintain procedural consistency with previous studies, but were not used for analyses. Data is available for STAI-State scores, and could be explored e.g., to assess hypotheses regarding state-dependent effects of the intervention (11). For the main outcome variables, we used Bayesian regression models and a parameter estimation approach, using *brms* (32) in *R* (33). All models used weakly informative priors (priors that do not favor any direction for an effect but constrain the model to sample from less extreme values than if a flat prior was given). Full details on each model, including regression formulae and the priors used, are provided in Supplementary Material section Regression Model Specification and Priors.

As parameter estimates for regressions on each key outcome variable, we provide a point estimate for the parameter (the mean of the posterior distribution) and a 95% highest density interval (HDI). The lower and upper HDI reflect the lower and upper bounds of the 95% most probable values in the posterior distribution for that parameter. Though not exactly the same, the 95% HDI can be considered as similar to two-sided 95% confidence intervals, meaning that if the HDIs fully exclude 0, it indicates that the respective variable is likely having a non-negligible effect on the outcome variable.

#### BAT Scores

For the BAT, cumulative ordinal regression with a probit link was used to compare the BAT scores achieved in each fear reactivation group. Full explanation of cumulative regression is beyond the scope of this paper, but an accessible tutorial is available (34). The cumulative probit regression models BAT scores as arising from a latent normal distribution with a mean of 0 and standard deviation of 1 (i.e., z-scores). It assigns cut-points along the normal distribution, which represent the different possible ordinal values and the proportion of participants estimated to have that response (e.g., if Cutpoint 1 is −1, this corresponds to a cumulative density on the standard normal distribution of approximately 0.16, meaning that on average, 16% of participants would be expected to score 1). The cutpoints function like the intercept in typical regression, indicating the cutpoints for a reference condition (in our case, the *Standard* fear reactivation condition). Other regression parameters in the model represent a shift in these cutpoints on the latent scale, thereby increasing or decreasing the proportion of people attaining higher or lower BAT scores. Negative parameter values indicate an increased probability of *low* scores, and positive values indicate an increased probability of *high* scores.

#### Distress and SPQ Scores

For Distress scores and Long-term SPQ scores (6- and 14-month follow-up), multilevel regression was used. Timepoint (for Distress, treatment session distress vs. post-treatment BAT distress; for Long-term SPQ scores, pre-treatment vs. 6 m post-treatment vs. 14 m post-treatment) and fear reactivation group were entered as interacting factors, and varying intercepts for each participant. For weekly SPQ follow-ups, multilevel models were run with weeks since treatment as a continuous variable, interacting with fear reactivation group as a factor, and varying intercepts for each participant.

#### Physiological Measures and Baseline Sample Characteristics

Physiological measures were assessed using repeated measures Bayesian ANOVAs, with Bayes Factors obtained using the default priors in *JASP* (35). These ANOVAs included fear reactivation group (Standard vs. Observe vs. Enclosure vs. Loose Tarantula) and timepoint (pre- vs. post-propranolol) as factors, with their interaction also assessed. Several additional analyses were run in *JASP* to assess possible confounds across groups on baseline questionnaire variables (ANOVAs or *t*-tests to compare groups on baseline questionnaire scores, Kendall's tau correlation coefficients to determine possible confounding relationships among baseline questionnaire scores and key outcome variables, and contingency tests to compare gender distributions across conditions). Full output from these analyses is provided in Supplementary Material sections Assessment of Confounds, ANOVAs for Physiological Measures, ANOVAs and Contingency Tables for Baseline Measures, and Long-Term Follow-Ups of Experiment 2.

Bayes Factors provide a ratio of evidence for or against a null hypothesis, with values below 1 indicating evidence in favor of the null, values above 1 indicating evidence in favor of the alternative hypothesis (i.e., differences between groups or the presence of a correlation), and values of approximately 1 providing equivocal evidence. It can be more difficult to provide convincing evidence *for* the null than against it, and so we take a conservative approach in interpreting Bayes Factors for an effect: Bayes Factors of <3 are typically considered weak evidence, from 3 to 10 are considered moderate evidence, and 10 or more considered strong. Bayes Factors of 100 or more are considered overwhelming evidence for the effect in question (36).

## Results

### Experiment 1

#### Baseline Characteristics

The sample was predominantly female. Across fear reactivation groups, there was no evidence for differences in baseline SPQ, PHQ, or STAI-T scores (or there was evidence against such differences), with slight evidence for differences in ASI scores, which tended to be slightly lower in the Standard and Observe groups than in the Enclosure and Loose Tarantula groups. Correlations were run to determine whether baseline SPQ, ASI, PHQ, STAI-T, or Age might be predictive of change over time in, or baseline assessments of, key variables in the analyses below (Supplementary Material section ANOVAs and Contingency Tables for Baseline Measures). There was no evidence for such possible confounding influences, and so these baseline scores were not included in the regression models below.

#### Physiological Analyses

Heart rate, diastolic blood pressure, and systolic blood pressure were assessed via means of three Bayesian mixed measures ANOVAs using default prior specifications in *JASP*, with group (between subjects: standard, observe, enclosure, loose tarantula) and timepoint (within subjects: pre- vs. post-propranolol) as factors. For systolic blood pressure and heart rate, *BF*_*Inclusion*_ values indicated overwhelming support for a decrease in scores over time (Heart rate *BF*_*Inclusion*_ = 2.913e+6; Systolic BP *BF*_*Inclusion*_ = 8.289e+10), whereas there was evidence against, or equivocal evidence regarding, effects of group (HR *BF*_*Inclusion*_ = 0.473, Systolic BP *BF*_*Inclusion*_ = 0.446) or a group ^*^ timepoint interaction (HR *BF*_*Inclusion*_ = 0.728, Systolic BP *BF*_*Inclusion*_ = 0.368). Hence, heart rate and systolic blood pressure were seen to decrease after receiving propranolol irrespective of condition. For diastolic blood pressure, *BF*_*Inclusion*_ values suggested equivocal evidence for an effect of time (*BF*_*Inclusion*_ = 1.033), and equivocal evidence or evidence against any group (*BF*_*Inclusion*_ = 0.598) or group ^*^ timepoint interaction (*BF*_*Inclusion*_ = 0.655). Group means over time and full output can be found in Supplementary Material section ANOVAs for Physiological Measures.

#### Behavior and Emotional Response to BAT

**Figure 2:**
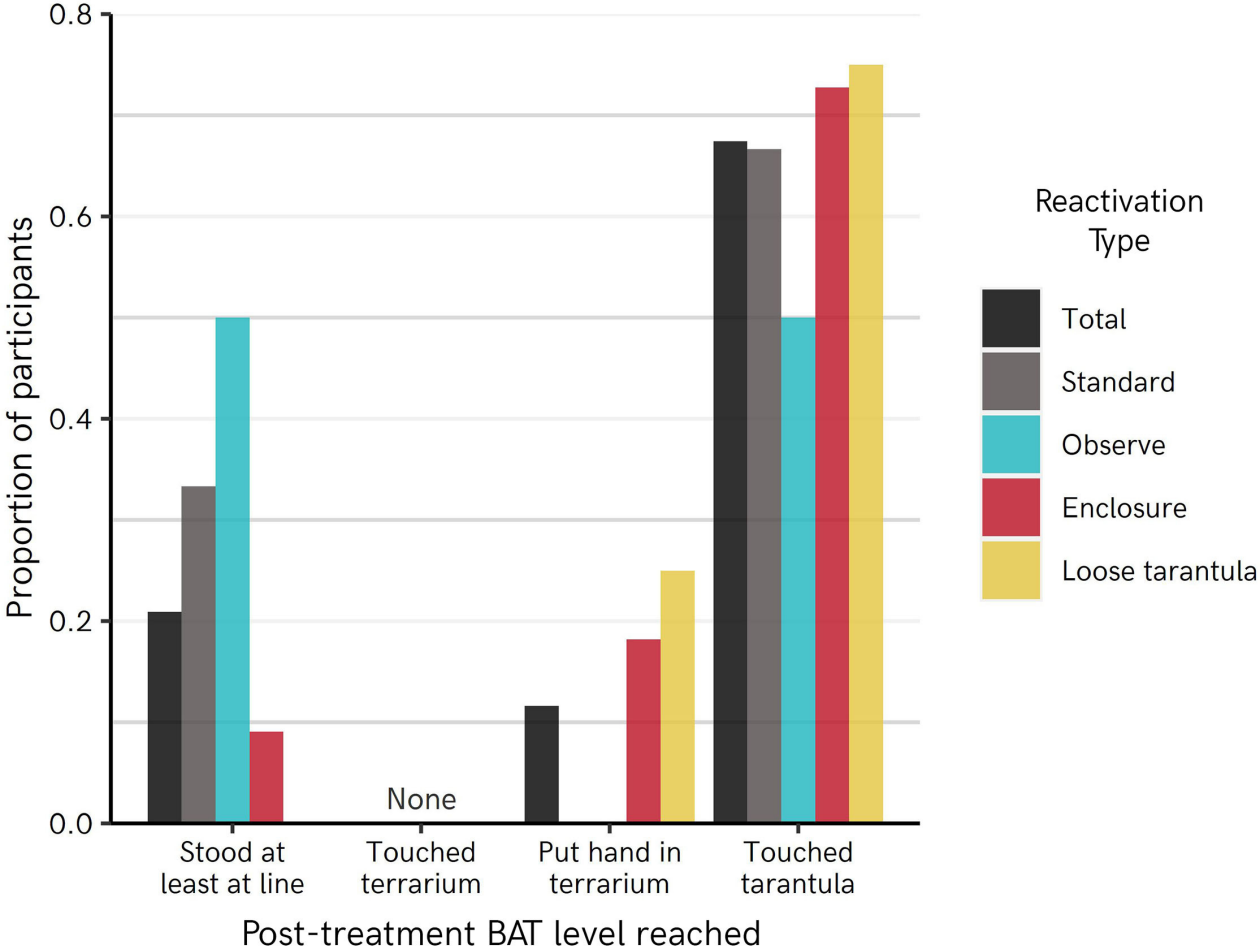
Post-treatment behavioral approach test (BAT) scores achieved by participants across different groups in Experiment 1.

Figure 2 shows raw proportions of participants per reactivation group who reached each step of the BAT. Qualitatively, the Observe group performed the poorest, with 50% of participants not going far enough to touch or reach into the terrarium, and the other 50% touching the tarantula. In other groups, between 67 and 75% of participants were able to touch the tarantula. A cumulative ordinal regression model predicting BAT score from fear reactivation group (Table 2) similarly indicated a slight tendency for the Observe group to perform marginally worse relative to other groups (Observe vs. Standard = −0.46 [−1.36–0.49] SDs on latent scale). However, no group was reliably better or worse than the Standard group (Loose Tarantula = 0.51 [−0.40–1.37], Enclosure = 0.35 [−0.54–1.25]).

**Table 2:** Regression parameter estimates for BAT scores in Experiment 1.

**Parameter**	**Mean**	**SD**	**Lower 95% HDI**	**Upper 95% HDI**	**Rhat**	**Bulk ESS**	**Tail ESS**
Cutpoint 1^*^	−0.78	0.33	−1.41	−0.12	1	14,686	15,995
Cutpoint 2^*^	−0.7	0.33	−1.34	−0.05	1	15,117	16,887
Cutpoint 3^*^	−0.27	0.32	−0.9	0.34	1	16,902	17,781
Enclosure	0.35	0.46	−0.54	1.25	1	16,846	16,667
Loose tarantula	0.51	0.45	−0.4	1.37	1	15,820	16,071
Observe	−0.46	0.47	−1.36	0.49	1	17,738	15,694

*SD, standard deviation; HDI, highest density interval; ESS, effective sample size*.

* *Cutpoints are determined with reference to the Standard fear reactivation condition*.

**Figure 3:**
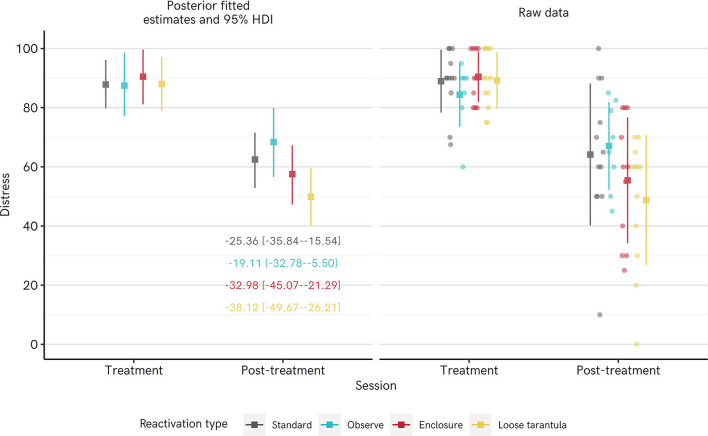
Distress scores when confronted with a spider dropped from the treatment session to the post-treatment session in Experiment 1. Left panel shows mean posterior parameter estimates from a Bayesian regression model (square points) and their 95% highest density intervals (HDI: whiskers), and mean difference scores [95% HDIs] as text. Right panel shows the raw data points (round points), with the mean (square points) ±1 standard deviation (whiskers).

Distress scores measured upon exposure to the spider at the treatment vs. post-treatment session indicated that all groups showed statistically reliable reductions in distress (Table 3). Note that for the Enclosure group, the distress scores from the *enclosure*, rather than tarantula BAT are taken. When the tarantula BAT distress is taken as the follow-up assessment, there is still a drop of −19.92 [−31.35 to −8.97]: model output in Supplementary Material section Additional Regression Model Output). Figure 3 shows both the raw data and fitted means from the posterior distribution of a Bayesian regression model. The largest difference was for the Loose Tarantula group, with an average estimated drop of −38.21 [−49.80 to −26.60] points, whereas the smallest difference was for the Observe group (−19.46 [−32.84 to −6.06]), matching the trend in BAT performance. Reliable reductions in distress remain when modeling the data using a *t* rather than a normal distribution, which is less influenced by extreme values (see Supplementary Material section Additional Regression Model Output).

**Table 3:** Regression parameter estimates for distress scores in Experiment 1.

**Parameter**	**Mean**	**SD**	**Lower 95% HDI**	**Upper 95% HDI**	**Rhat**	**Bulk ESS**	**Tail ESS**
Intercept (standard)	88.99	3.92	81.42	96.77	1	19,634	18,860
Enclosure	0.72	5.58	−10.14	11.75	1	21,657	19,668
Loose tarantula	−0.63	5.48	−11.28	10.12	1	21,851	17,089
Observe	−2.9	5.95	−14.61	8.85	1	24,085	17,920
Post-treatment	−24.89	5.16	−34.97	−14.74	1	18,076	18,518
Enclosure, post-treatment	−8.28	7.3	−22.71	6.12	1	21,689	18,499
Loose tarantula, post-treatment	−13.32	7.35	−27.83	0.95	1	20,502	17,925
Observe, post-treatment	5.44	7.66	−10.14	20.24	1	22,976	20,022
SD of ppn intercept	5.39	3.02	0	10.4	1	5,191	8,414
Sigma	15.7	1.53	12.69	18.66	1	10,899	15,032

*SD, standard deviation; HDI, highest density interval; ESS, Effective sample size*.

#### Follow-Up Questionnaire Data

Multilevel regression was used to analyze SPQ data from baseline, the post-treatment session, and subsequent weekly follow-ups. Separate regressions were run with a simple linear effect of time since treatment, and with a second order polynomial effect of time (weeks^2^). Assessment using Widely Applicable Information Criterion (WAIC) favored the polynomial model as better representing the data (vs. Polynomial model, Linear model WAIC ELPD-difference = −65.1, SE = 12.4). Hence, we present the results of the polynomial model below (linear model output is present in Supplementary Material section Additional Regression Model Output).

**Figure 4:**
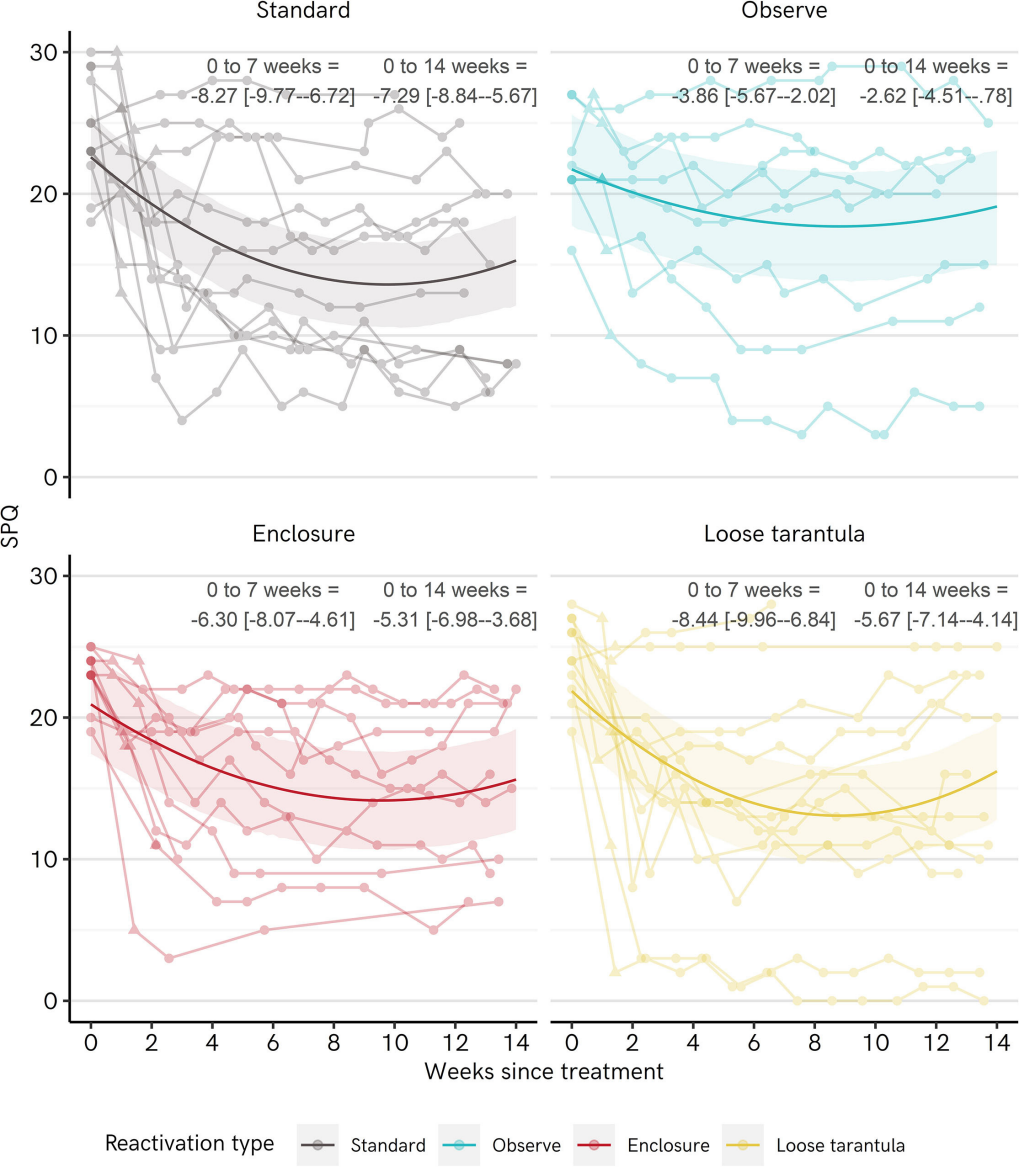
Change in spider phobia questionnaire (SPQ) scores over time for each group in Experiment 1. The dark lines and ribbons represent fitted point estimates and 95% highest density intervals (HDIs) from the regression model. Points along faded lines represent responses of individual participants. The triangular point designates the score measured in the post-treatment session. 0–7 and 0–14 weeks = change from pre-treatment to 7- and 14-weeks post-treatment, respectively, with point estimates and [95% HDIs].

Table 4 shows the regression output and Figure 4 shows fitted regression model estimates and raw data for SPQ scores over time in each group. In each group there is a general slope downwards, which levels out or slightly turns upwards, over time. Notably, the decline in the Observe group appears slightly shallower than the other groups. This is evident from the parameter estimate for the interaction between the Observe group and the linear effect of time since treatment, which partially cancels out the drop in scores observed in the Standard group. The fitted regression estimates indicate that on average, SPQ scores drop in each group from 0 to 7 weeks post-treatment and from 0 to 14 weeks post-treatment (see the numbers in Figure 4). The final estimate for change is lowest for the Observe group, with change scores being reliably, if only slightly, higher in all other groups (vs. Standard = 4.67 [2.30–7.18], vs. Loose Tarantula = 3.05 [0.59–5.40], vs. Enclosure = 2.69 [0.21–5.19]).

**Table 4:** Regression parameter estimates for weekly SPQ scores in Experiment 1, quadratic model.

**Parameter**	**Mean**	**SD**	**Lower 95% HDI**	**Upper 95% HDI**	**Rhat**	**Bulk ESS**	**Tail ESS**
Intercept	22.59	1.53	19.56	25.57	1	5,117	7,104
Enclosure	−1.65	2.13	−5.86	2.59	1	5,766	8,414
Loose tarantula	−0.71	2.13	−4.84	3.52	1	6,233	8,357
Observe	−0.86	2.3	−5.33	3.73	1	5,871	8,956
Weeks	−1.84	0.2	−2.23	−1.44	1	7,442	11,450
Weeks 2	0.09	0.01	0.07	0.12	1	7,515	10,389
Enclosure, weeks	0.42	0.3	−0.17	1.01	1	8,913	11,983
Loose tarantula, weeks	−0.17	0.28	−0.72	0.39	1	8,774	11,766
Observe, weeks	0.93	0.31	0.31	1.53	1	8,897	11,241
Enclosure, weeks 2	−0.02	0.02	−0.06	0.02	1	8,804	12,244
Loose tarantula, weeks 2	0.02	0.02	−0.02	0.06	1	8,820	12,240
Observe, weeks 2	−0.04	0.02	−0.08	0	1	8,943	11,825
SD of ppn intercept	6.13	0.71	4.93	7.71	1	4,106	6,145
Sigma	2.98	0.1	2.8	3.18	1	16,683	15,479

*SD, standard deviation; HDI, highest density interval; ESS, effective sample size*.

One further multilevel regression, presented in full in the Supplementary Material section Long-Term Follow-Ups in Experiment 1 assessed changes from pre-treatment SPQ scores to scores at 6- and 14-month follow-up, among participants who provided either of these follow-up measures. Reductions in SPQ scores from pre-treatment were apparent approximately 6- and 14-months post-treatment in the Standard and Loose Tarantula groups. Reliable reductions persisting to 14 months post-treatment were not evident for the Enclosure or Observe groups. However, it should be noted that there were only a handful of respondents remaining at 14 months (6 m Standard *n* = 7, Observe *n* = 7, Loose tarantula *n* = 5, Enclosure *n* = 5; 14 m Standard *n* = 7, Observe *n* = 3, Loose tarantula *n* = 7, Enclosure *n* = 4. Six participants at 6 months and one at 14 months were excluded for not completing the questionnaire within a reasonable time from the intended follow-up date, and one 6-month participant was mistakenly given the questionnaire 20 days early—a regression including these participants yielded comparable results).

### Experiment 2—Methods

#### Experiment 2

After discussing participants' experiences of the treatment, observing their behavior, and seeing the early-stage results across groups in Experiment 1, we suspected that the treatment might not have been operating similarly to how it had been in Soeter and Kindt (21). A primary concern was that adaptations of the procedure to accommodate the clinical participants might have increased the chance of non-specific effects such as placebo responding or exposure driving observed changes, rather than a specific effect of reactivation with propranolol. If this were the case, then the findings would not inform us as to optimal “reconsolidation-based” approaches to treatment. We therefore undertook a double-blind, placebo-controlled re-run of the Standard procedure. Formal power assessments were not performed because the effect was so strong in Soeter and Kindt (21) as to be essentially binary, with no effect in the placebo group and a very large effect in the propranolol group. We aimed for a comparable sample size to Soeter and Kindt (21) of 15 participants per group, concluding with 13 per group due to practical constraints on project completion. This sample size may be limited for drawing strong conclusions about effect sizes or the presence of small group differences, but was sufficient for confirming whether placebo effects might just as likely explain post-treatment behavior as a true intervention effect.

#### Recruitment and Exclusion Criteria

Inclusion/exclusion criteria were the same as in Experiment 1. Thirty-six participants came to intake, of whom 26 completed all sessions and are included in analyses below. Reasons for exclusion were: not responding to contact/dropping out before completing all sessions (*n* = 3), low heart rate (*n* = 1), presenting as subclinical at interview (*n* = 3), and not receiving approval from their doctor (*n* = 3). Table 5 shows sample sizes and descriptive statistics for each group in Experiment 2, with statistical results presented in the Experiment 2 Results section Baseline Characteristics.

**Table 5:** Baseline characteristics of placebo and propranolol participants.

		**Mean**	**SD**	**Min**	**Max**	***BF_***Condition***_***	***p* condition**
Age	Placebo	28.85	10.46	19	54	0.384	0.701
	Propranolol	27.54	6.12	19	42		
SPQ	Placebo	24.15	3.98	16	30	0.485	0.384
	Propranolol	22.92	3.04	18	27		
ASI	Placebo	7.77	5.29	2	19	0.580	0.269
	Propranolol	10.15	5.46	3	22		
PHQ	Placebo	2.69	1.49	1	6	0.636	0.227
	Propranolol	3.46	1.66	1	6		
STAIT	Placebo	34.65	7.61	25	51	0.420	0.537
	Propranolol	36.42	6.76	26	49		
						**Contingency:**	
		**M:F**				***Bayes Factor***	***p*** **-value**
Sex	Placebo	1:12				0.944	0.227
	Propranolol	3:10					

*BF_Condition_, Bayes factor for inclusion of condition in ANOVA; p condition, p-value for difference between groups in ANOVA*.

### Materials and Measures

#### Propranolol

Propranolol administration was the same as in Experiment 1, with the exception that the researcher and clinician did not know whether placebo or propranolol was being administered (double-blind), and there was a 50% chance of the participant receiving placebo. A member of the lab support placed each pill into an envelope shortly before the treatment session and gave it to the clinician. Analyses were performed unblinded.

#### Tarantula Behavioral Approach Test

As in Experiment 1.

#### Standardized Questionnaires

The same questionnaires as in Experiment 1 were used. In addition, at 2 and 6 months post-treatment, we gave participants a modified SPQ scale, with a six-point Likert scale format (from Strongly Disagree to Strongly Agree), rather than binary responses, and the FSQ.

### Procedure

**Figure 5:**
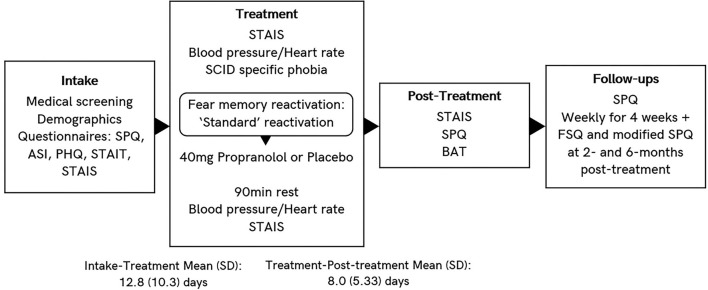
Schematic overview of Experiment 2.

Figure 5 provides a schematic overview of the procedure for Experiment 2, and when different measures were obtained.

#### Intake Session

As in Experiment 1.

#### Treatment Session

Treatment sessions took place on average 13 days after the intake session. Most (18) sessions took place within 14 days of intake, but eight sessions occurred between 15 and 32 days. The treatment protocol was the same as the Standard group in Experiment 1, except with the possibility that the patient received placebo rather than propranolol. The duration of exposure for each group is presented in Supplementary Material section Duration of Exposure at Treatment, with analyses suggesting no evidence of differences between groups.

#### Post-treatment Session

As in Experiment 1, except that the pill the participant received (Placebo vs. Propranolol) was not known to the experimenter and was not revealed to the participant until they had completed a 2-month follow-up questionnaire. Post-treatment sessions occurred on average 8 days after treatment, and all but two participants returned within 10 days since treatment. The remaining two participants did their post-treatment sessions 19 and 29 days after the treatment session.

#### Questionnaire Follow-Up

Participants were followed up with weekly SPQ questionnaires for a 4-week period, starting from the post-treatment session. We also attempted to reach participants 2 and 6 months after treatment session, using the FSQ and the modified SPQ. The condition was revealed to participants at 2-month follow-up.

### Analytic Approach

The analytic approach was the same as in Experiment 1, with the exception that for the longer-term follow-ups (2- and 6-months post-treatment), different questionnaires were used than at baseline, preventing comparison to pre-treatment. These questionnaires were simply analyzed using Bayesian ANOVAs with default priors from *JASP* to compare Placebo vs. Propranolol groups.

## Results

### Experiment 2

#### Baseline Characteristics

The sample was again predominantly female, and the placebo and propranolol groups showed no evidence of baseline differences in age, nor in SPQ, ASI, PHQ, or STAIT scores (Table 5). In one assessment of possible confounds (Supplementary Material section Assessment of Confounds), ASI scores were slightly related to BAT scores, and so z-transformed ASI scores were included in the regression for BAT scores below.

#### Physiological Analyses

Heart rate, diastolic blood pressure, and systolic blood pressure were analyzed using three Bayesian mixed measures ANOVAs with *JASP*'s default prior specifications, with Group (Between subjects: Placebo vs. Propranolol) and Timepoint (Within subjects: pre- vs. post-propranolol) as factors. Both systolic blood pressure and heart rate showed overwhelming evidence in favor of change over time, with scores dropping from before vs. after receiving propranolol (Heart rate *BF*_*Inclusion*_ = 1.893e +6; Systolic BP *BF*_*Inclusion*_ = 15107.699). However, support for a Timepoint ^*^ Group interaction was equivocal for systolic blood pressure (*BF*_*Inclusion*_ = 0.942) and still quite weak for heart rate (*BF*_*Inclusion*_ = 2.740, indicating a greater decrease in the propranolol group). There was weak evidence against, or equivocal evidence, regarding a group effect (Heart rate *BF*_*Inclusion*_ = 0.967; Systolic BP *BF*_*Inclusion*_ = 0.622). Hence, heart rate and systolic blood pressure were seen to decrease from the start of the treatment session to the end, after having received a pill, but there was only quite weak evidence that these reductions were greater among propranolol than placebo participants. For diastolic blood pressure, *BF*_*Inclusion*_ values suggested equivocal evidence for an effect of time (*BF*_*Inclusion*_ = 1.113), and slight evidence against any effect of group (*BF*_*Inclusion*_ = 0.505) or Group ^*^ Timepoint interaction (*BF*_*Inclusion*_ = 0.344). Full output and group means over time can be found in Supplementary Material section ANOVAs for Physiological Measures.

#### Behavior and Emotional Response to BAT

**Figure 6:**
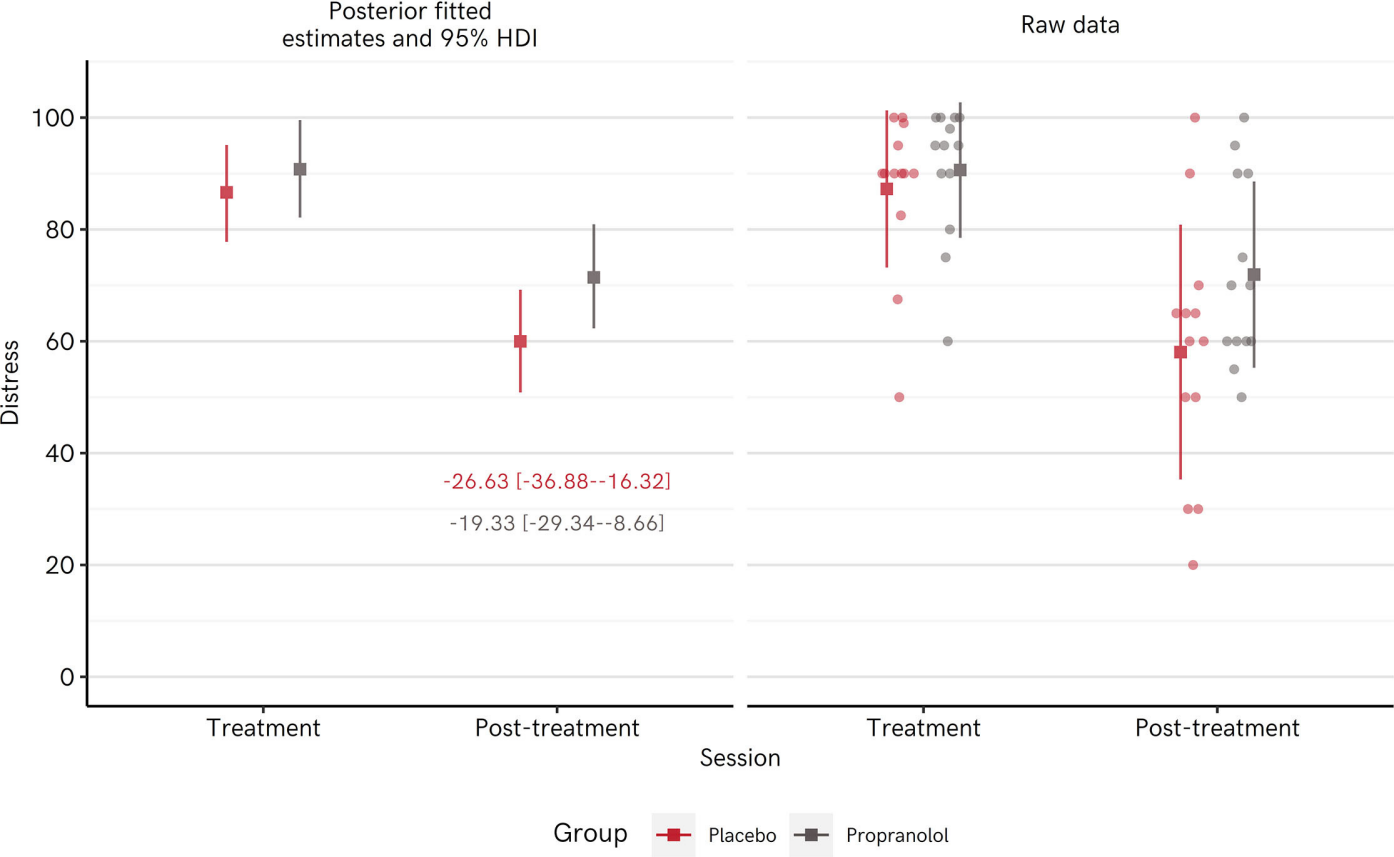
Distress scores when confronted with a spider dropped from the treatment session to the post-treatment session in Experiment 2. Left panel shows mean posterior parameter estimates from a Bayesian regression model (square points) and their 95% highest density intervals (HDI: whiskers), and mean difference scores [95% HDIs] as text. Right panel shows the raw data points (round points), with the mean (square points) ±1 standard deviation (whiskers).

Figure 6 depicts raw proportions of participants per condition who reached each step of the BAT. Qualitatively, the Propranolol participants performed marginally worse than Placebo participants, with 46% touching the tarantula (vs. 62% in the placebo condition). Parameter estimates from a cumulative ordinal regression model determining BAT score from group, and incorporating possible influence of ASI scores, did not reliably favor Placebo or Propranolol (Propranolol = −0.30 [−1.13–0.55] in SDs on the latent scale, vs. Placebo). Z-scored ASI scores were related to slightly lower BAT performance −0.51 [−0.98 to −0.04] (Table 6).

**Table 6:** Regression parameter estimates for BAT scores in Experiment 2.

**Parameter**	**Mean**	**SD**	**Lower 95% HDI**	**Upper 95% HDI**	**Rhat**	**Bulk ESS**	**Tail ESS**
Cutpoint 1^*^	−1.23	0.38	−1.96	−0.45	1	15,090	14,924
Cutpoint 2^*^	−0.74	0.36	−1.43	−0.04	1	18,559	17,965
Cutpoint 3^*^	−0.2	0.34	−0.87	0.44	1	24,263	18,905
Propranolol	−0.3	0.43	−1.13	0.55	1	18,979	15,566
ASI z-scored	−0.51	0.24	−0.98	−0.04	1	16,824	15,067

*SD, standard deviation; HDI, highest density interval; ESS, effective sample size*.

* *Cutpoints made with reference to the Placebo condition*.

Distress scores measured upon exposure to the spider at treatment session vs. post-treatment session showed reliable reductions in distress in both groups, though again the reduction in the Placebo group was qualitatively greater than in the Propranolol group. Figure 6 shows both the raw data and fitted means from the posterior distribution of a Bayesian regression model (full model in Table 7). The largest difference was for the Placebo group, with an average estimated drop of −26.63 [−36.88 to −16.32] points, whereas the drop in the Propranolol group was −19.33 [−29.34 to −8.66] points, paralleling the trend for poorer BAT performance in the propranolol group.

**Table 7:** Regression parameter estimates for distress scores in Experiment 2.

**Parameter**	**Mean**	**SD**	**Lower 95% HDI**	**Upper 95% HDI**	**Rhat**	**Bulk ESS**	**Tail ESS**
Intercept	86.62	4.42	77.77	95.1	1	16,288	17,086
Propranolol	4.13	5.84	−7.46	15.44	1	16,452	16,858
Post-treatment	−26.63	5.18	−36.88	−16.32	1	21,187	18,036
Propranolol, post-treatment	7.29	6.77	−6.1	20.64	1	19,901	18,393
SD of ppn intercept	9.27	3.75	0.8	15.84	1	4,291	5,289
Sigma	14.31	2.16	10.31	18.5	1	6,414	13,103

*SD, standard deviation; HDI, highest density interval; ESS, effective sample size*.

#### Follow-Up Questionnaire Data

Multilevel regression was again used for analyzing SPQ data from the post-treatment session and subsequent weekly follow-ups. In contrast to Experiment 2, adding a second-degree polynomial for time since treatment gave no indication of better fit to the data vs. a linear effect of time (vs. Linear model, Polynomial model WAIC ELPD-difference = −0.5, SE = 2.3). This is probably due to the shorter time frame of weekly follow-ups primarily capturing the initial drops in SPQ scores but not the more prolonged leveling out over greater durations. We therefore present the results of the simple linear model below.

**Figure 7:**
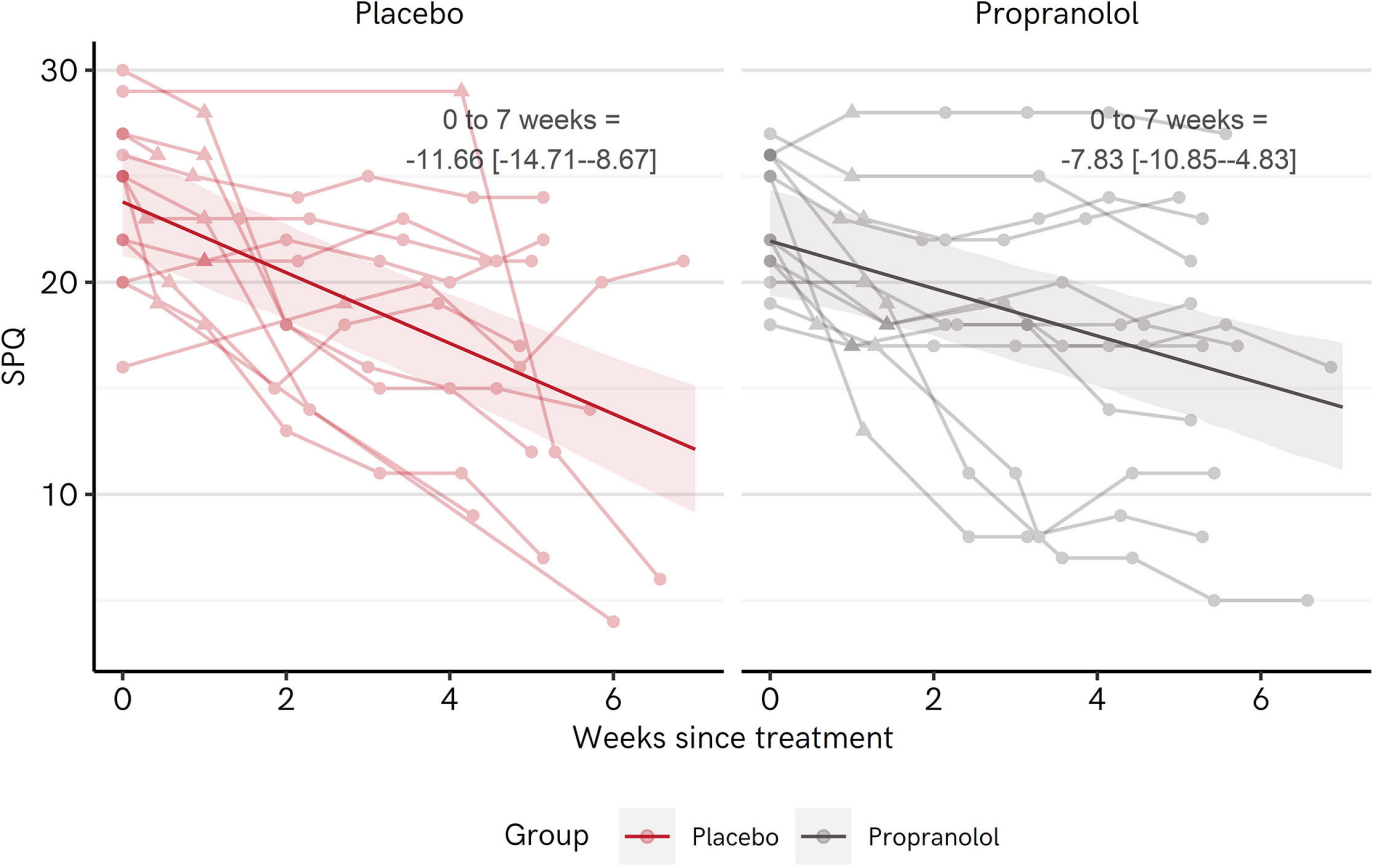
Change in spider phobia questionnaire (SPQ) scores over time for each group in Experiment 2. The dark lines and ribbons represent fitted point estimates and 95% highest density intervals (HDIs) from the regression model. Points along faded lines represent responses of individual participants. The triangular point designates the score measured in the post-treatment session. 0–7 weeks = change from pre-treatment to 7 weeks post-treatment, respectively, with point estimate and [95% HDI].

Table 8 shows the regression output and Figure 7 shows fitted regression model estimates and raw data for SPQ scores over time for Placebo vs. Propranolol. For both Placebo and Propranolol groups, SPQ scores tended to decrease from pre- to post-treatment. The parameter estimate for Propranolol ^*^ Time interaction is tending in a positive direction. Change over time in the Propranolol group is thus very slightly less in the Propranolol than the placebo group when estimated at 7 weeks post-treatment (vs. Placebo change = −3.83 [−7.93 −0.61]).

**Table 8:** Regression parameter estimates for weekly SPQ scores in Experiment 2, linear model.

**Parameter**	**Mean**	**SD**	**Lower 95% HDI**	**Upper 95% HDI**	**Rhat**	**Bulk ESS**	**Tail ESS**
Intercept	23.79	1.25	21.25	26.18	1	5,870	10,470
Propranolol	−1.84	1.69	−5.18	1.47	1	6,035	9,682
Weeks	−1.67	0.22	−2.1	−1.24	1	12,905	16,026
Propranolol, weeks	0.55	0.31	−0.09	1.13	1	12,445	14,878
SD of ppn intercept	3.94	0.7	2.65	5.34	1	5,614	9,369
sigma	3.62	0.24	3.16	4.11	1	17,182	16,071

*SD, standard deviation; HDI, highest density interval; ESS, effective sample size*.

After observing some of the incoming SPQ scores, we considered whether it might be possible that the True/False format of the SPQ could obscure some group differences. For example, it is possible that in the Placebo group, participants were actively looking for changes and therefore inclined to disagree with SPQ items even if only slightly changing since before treatment, whereas in the Propranolol group, effects might be stronger but could also only be indicated by selecting the binary option in the same direction as the Placebo participants. We reached out to participants again 2 and 6 months after treatment with a modified version of the SPQ in which a greater range of Likert scale responses were possible, and the FSQ, which is also based on a Likert scale. Exploratory pairwise comparisons presented in Supplementary Material section Long-Term Follow-Ups in Experiment 2 indicated at most very weak evidence for lower FSQ scores among *placebo* participants (Bayes Factors of between 1.04 and 1.08, *p* > 0.1). All other Bayes Factors were <1, with corresponding *p*-values > 0.1, suggesting no evidence for group differences, or evidence against group differences in questionnaire scores at approximately 2- and 6-months post-treatment. Hence, there was no support for the idea that improvement in the Propranolol group might have been hidden by the binary SPQ response options.

## Discussion

We found that undergoing a reconsolidation-based procedure for arachnophobia typically reduced participants' fear of spiders from pre- to post-treatment. For some participants, changes in self-reported fear were striking and very large, and differences relative to pre-treatment tended to still be present even 6-months or over a year after the treatment session. However, some participants' fear appeared generally unchanged relative to pre-treatment, with SPQ scores relatively constant across time. One interpretation could be that the conditions for triggering and interfering with reconsolidation were met among some participants, but not among others, due to some unspecified boundary conditions. This would cause a split among the participants, whereby some experience considerable changes in their fear, and others remain mostly stable. However, this interpretation is undermined by observations from Experiment 2, which suggested that a group receiving placebo showed just as much benefit as those who received propranolol. A parsimonious explanation would therefore be that across the reactivation procedures we investigated, changes in fear of spiders were not attributable to interference with reconsolidation, but to non-specific factors, such as expectation/placebo effects and exposure. Given the brevity of the intervention tested here relative to typical therapies, the presence of quite strong non-specific effects may be seen to demand more explanation than if we had simply observed *no* effects in the procedures we tested.

Differences between the current study and our previous intervention with subclinical participants are present at both the design and participant level, and may help explain some differences in the observed outcomes. Previously (21), participants performed some behavioral tests with spiders before the treatment session, and were excluded if they reached too high a level of performance. We did not perform such pre-tests here because, in piloting interventions with clinical patients previously, we found that they struggled to differentiate between “measurement” and “treatment” sessions. Despite instructions, many patients would push (under considerable duress) to get the maximum score on the behavioral tests because they felt it would help them overcome their fear. This caused genuinely fearful patients to be excluded. By not including this step, it is possible that some participants would already have been able to perform well in the tarantula BAT even before treatment. However, this seems unlikely given the clinical presentation of the participants, and that some participants were excluded at treatment owing to subclinical fear levels revealed during the treatment session.

One further consideration is that, since Soeter and Kindt (21), there has been increased media attention regarding reconsolidation-based treatments, including in Dutch news. Although we did not record the frequency of this, many patients reported first hearing about the research through news broadcasts, and were also able to find coverage of the treatment in online media outlets when doing research to decide whether or not to sign up. In addition, participants were presented with a richer rationale for the treatment in the present experiment, including information about how it had worked previously with spider fearful individuals. In Experiment 1, participants were also told after the post-treatment session that they had received propranolol. Such factors may have increased the potential for non-specific effects to occur, for example owing to increased expectation that the treatment would work after seeing positive news coverage. We only have a very rough measure of patients' trust in the procedure—a single Likert-scale item obtained at the end of the intake session—and exploratory analyses of this item did not suggest that it was correlated with any outcomes in the experiment (see Supplementary Material section Trust in Treatment). However, the measure was taken before contact with the clinician in the experiment, who provided additional explanation of the procedure, and may have increased patients' confidence in the treatment after this measure was taken. It would be of interest to gather more fine-grained assessments of patients' faith in the treatment they are receiving in future studies.

It is worth considering the extent to which simply believing that one will not experience fear as a result of a reconsolidation-based intervention may free fearful individuals from some of the most concerning aspects of their phobias. Fearful individuals anticipate feeling highly unpleasant anxiety responses upon confrontation with feared objects/situations, which feature prominently in their concerns about their feared stimuli (37) and may cause avoidance and anxiety through “fear of fear” (29). Belief in the treatment may have reduced such expectations during the test session and better enabled patients to re-evaluate their anxiety responses and capabilities, in turn improving their confidence when outside of the study sessions. Additionally, we encouraged participants to “test out” their fear levels after treatment, potentially providing further opportunity and impetus for fear re-evaluation, as patients were likely looking for changes relative to pre-treatment. The post-treatment session also provided participants with the opportunity to cement any gains they noticed, as all patients were shown what happened when the researcher touched the tarantula, and some opted to continue observing and interacting with it for a short time after the official BAT was completed.

One final key difference is that patients' treatment sessions were somewhat longer in the present study than in Soeter and Kindt (21). In preparation for their fear reactivation, participants underwent a more in-depth discussion with the clinician (second author) about their fear and its history. Additionally, the exposure itself typically lasted longer than the 2-min reactivation protocol used in Soeter and Kindt (21), as most patients needed more encouragement and time to be able to approach the tarantula in the present study. Reactivation-dependent amnesia has been found to be highly sensitive to the duration of reactivation, or to the amount of novel information that can be conveyed with different reactivation lengths (38, 39). It is possible that the extent of reactivation in the present study was not optimal for inducing reconsolidation, instead tipping patients toward extinction learning or a “limbo” phase (40), in which neither reconsolidation nor extinction are triggered.

These considerations present difficulties for the clinical translation of reconsolidation-based interventions. If reconsolidation-like effects are highly sensitive to exactly how fear is reactivated, it may prove difficult to establish the optimal means of reactivation for each patient. A generic form of reactivation that works for most people—as in Soeter and Kindt (21)—would be ideal, but it is possible that careful adjustments would need to be made for each person given idiosyncrasies of their fear, learning history, and temperament [see (41)]. With clinical patients seeking and expecting amelioration of their fears, rather than subclinical participants who are not actively seeking treatment, a greater degree of encouragement, guidance, and connection between patient and therapist is warranted. However, this may also increase the likelihood of non-specific effects or alter the experience of the reactivation session in a way that precludes the induction of reconsolidation. Clinicians will need to strike a balance between engaging the patient in their treatment sufficiently to undergo a fear-provoking reactivation, and simply administering exposure treatment as usual with the addition of a pill.

From a research perspective, our findings highlight the need for control conditions in reconsolidation-based clinical interventions. Evidently, some patients receiving placebo can experience quite strong reductions in their fear from even a very brief intervention. In some cases, effects in an uncontrolled intervention may be so striking and consistent that they can scarcely be explained by non-specific effects, but this is rare and still inconclusive. As just a few examples, control groups could include the use of placebo, amnesic agents delivered outside the “reconsolidation window,” or beta-adrenergic antagonists that do not cross the blood-brain barrier. With all the practical difficulties of clinical studies, researchers cannot be expected to close off *all* alternative explanations for observed effects in any one study, but comparison with some form of control seems advisable even where non-specific effects are not anticipated.

Notably, the presence of placebo/non-specific effects among arachnophobic patients here, and amongst participants undergoing a reconsolidation-based intervention for fear of public speaking in another recent study (20), does not point to non-specific effects as an explanation for previously observed effects of propranolol in reconsolidation-based studies. Soeter and Kindt (21) included controls for both non-specific effects of propranolol, and a placebo + reactivation control. In both these control groups, no improvement in fear of spiders was observed, even with repeated BATs with spiders. Moreover, placebo controls, as well as controls for different types of reactivation, have been used in a plethora of reconsolidation-based studies of human fear conditioning, again showing no effects in these control conditions [reviewed in (10)]. While previous successes of reconsolidation-based treatments highlight the best-case potential of such interventions, we do not have a strong handle on precisely how we can best translate such findings into full clinical interventions at this stage, and this may be substantially more difficult than previously expected. Some recent findings have even called into question the replicability of the basic laboratory phenomenon that such interventions are based on (42), demonstrating that the amnestic phenomena observed consistently in previous research are elusive and challenging to produce.

## Limitations

The present study is limited by the small sample sizes obtained, which constrain how certain we can be about potential group differences: small benefits in a particular group in Experiment 1 would likely not have been observed, and a small benefit for propranolol over placebo in Experiment 2 would likely also not be possible to detect with the present sample sizes. However, if anything there was a tendency for the placebo condition to perform *better* than the propranolol group, and we can also observe that patterns of change in SPQ scores over time were different from those observed in Soeter and Kindt (21), which took a longer time to become apparent. Hence, although we cannot rule out the possibility of some differences existing among the small-sized groups we tested, we can state that the pattern of results is not as expected and does not support an explanation of the findings based upon reconsolidation.

One further possible limitation is the dosage of propranolol used in the present experiment. Although 40 mg has been successful in previous studies—including those involving participants (21) with fear of spiders and patients with PTSD (18)—we cannot rule out that alternative dosing regimens might be necessary for some clinical conditions. We observed blood pressure and heart rate drops over time in both experiments 90 min post-propranolol. Again though, these drops were also observed in the placebo group and therefore could reflect relaxation effects from sitting down for 90 min as opposed to a strong propranolol effect. Hence, the possibility of suboptimal dosing of propranolol should not be completely ruled out.

## Conclusion

Our findings highlight the difficulties of translating reconsolidation-based experimental studies into clinical interventions. Striking changes in fear-related behavior and cognitions in studies such as Soeter and Kindt (21) show the prospect of reconsolidation-based interventions. If such potent effects can be harnessed in clinical interventions, this would a major development in the treatment of anxiety disorders. However, the present findings show that we do not yet fully understand how to optimally induce such effects, and further point to the value of controls for non-specific factors in reconsolidation-based treatments.

## Data Availability Statement

The datasets presented in this study can be found in online repositories. The names of the repository/repositories and accession number(s) can be found below: https://osf.io/2ay7f/?view_only=64fdf8d55e3b46d9a7348c02b4ddc4d4.

## Ethics Statement

The studies involving human participants were reviewed and approved by University of Amsterdam Institutional Review Board. The patients/participants provided their written informed consent to participate in this study.

## Author Contributions

JE and MK designed and performed the experiments. JE analyzed the data and wrote the manuscript. MK reviewed and made edits to the manuscript. Both authors contributed to the article and approved the submitted version.

## Funding

This work was supported by the ERC Advanced Grant 743263 of MK.

## Conflict of Interest

MK is a founder of Kindt Clinics, a phobia treatment facility in the Netherlands which uses reconsolidation-based treatment approaches. The remaining author declares that the research was conducted in the absence of any commercial or financial relationships that could be construed as a potential conflict of interest.

## Publisher's Note

All claims expressed in this article are solely those of the authors and do not necessarily represent those of their affiliated organizations, or those of the publisher, the editors and the reviewers. Any product that may be evaluated in this article, or claim that may be made by its manufacturer, is not guaranteed or endorsed by the publisher.
