# Supplementary material for: Placebo and Non-specific Effects in Reconsolidation-Based Treatment for Arachnophobia
Source: Front Psychiatry. 2021 Nov 12;12:775770. doi: 10.3389/fpsyt.2021.775770 (PMC8632940; doi:10.3389/fpsyt.2021.775770)
Supplement: Supplementary file 1 [file Data_Sheet_1.docx]

Supplementary materials for:

Placebo and non-specific effects in reconsolidation-based treatment for arachnophobia

Elsey, J. W. B., & Kindt, M.

**Medical inclusion/exclusion criteria for propranolol administration**

Participants underwent a medical screening to ensure they could safely take propranolol. In addition to receiving permission from their doctor, participants were screened for: heart conditions in first degree relatives; personal medical history of heart, circulatory, lung, liver, or kidney problems that would contraindicate the use of propranolol, use of contraindicated medications (e.g., medication that affect the heart/blood pressure); heart rate <55bpm before the treatment session, blood pressure <90/60; and pregnancy.

Table S1. Exclusions across studies.

| Study 1 |  |  | Study 2 |  |
| --- | --- | --- | --- | --- |
| Reason | *n* |  | Reason | *n* |
| Wrong spider | 2 |  | Wrong spider | 0 |
| Lost contact/dropped out | 7 |  | Lost contact/dropped out | 3 |
| HR | 3 |  | HR | 1 |
| Not following procedure | 2 |  | Not following procedure | 0 |
| Low fear of tarantula | 1 |  | Low fear of tarantula | 0 |
| Subclinical in interview | 0 |  | Subclinical in interview | 3 |
| No approval from doctor | 0 |  | No approval from doctor | 3 |
| Total | 15 |  | Total | 10 |
|  |  |  |  |  |
| Included | 43 |  | Included | 26 |

**Questionnaire Information**

*Spider Phobia Questionnaire (SPQ: Klorman et al., 1974).* The SPQ is a self-report questionnaire consisting of 31 statements indicative of fear of spiders, answered in a True/False format, indicating whether or not the statements apply to the respondent. Higher scores (0-31) indicate greater fear of spiders. The scale shows adequate test-reliability and internal consistency, and is responsive to changes as a result of treatment (Muris and Merckelbach, 1996).

*Modified SPQ.* A modified SPQ was used as an exploratory measure in some follow-up tests to determine whether some post-treatment changes might be due to participants wishing to indicate a small change in their fear, which would be limited by the True-False format of the SPQ. The questionnaire was the same as the SPQ, except using a 6-point Likert scale, from “Strongly Disagree” to “Strongly Agree”, with no middle option.

*Fear of Spiders Questionnaire (FSQ: Szymanski and O’Donohue, 1995).* The FSQ is a self-report questionnaire intended to assess fear of spiders. The scale has 18 items, to which participants respond on a 7-point Likert scale, from 1 (“Strongly Disagree”) to 7 (“Strongly Agree”). Higher scores (18-126) indicate greater fear of spiders. This scale also shows adequate test-reliability and internal consistency, and is responsive to changes as a result of treatment (Muris and Merckelbach, 1996).

*Spielberger State-Trait Anxiety Index (STAI: Spielberger, Gorsuch, & Lusthene, 1970).* The STAI is a self-report questionnaire, consisting of two 20-item subscales that measure state (STAI-S) and trait (STAI-T) anxiety. For the STAI-T, respondents answer how frequently they experience anxiety-related phenomena on a scale, from 1 ("almost never") to 4 ("almost always"). For the STAI-S, participants indicate how much they are currently experiencing feelings/thoughts related to anxiety, from 1 ("not at all") to 4 ("very much so"). The subscales have shown good internal consistency (ranging from .83-.92), and the STAI-T shows good test-retest reliability (*r* = .81) (Foa, Riggs, Dancu, & Rothbaum, 1993). A validated Dutch translation of the STAI was given to Dutch participants (van der Ploeg, 1980).

*Patient Health Questionnaire 9 (PHQ-9: Kroenke, Spitzer, & Williams, 2001).* The PHQ-9 is a 9 item self-report scale for assessing depression severity. Participants indicate how much they have experienced 9 depressive symptoms over the past 2 weeks, from 0 ("not at all"), to 3 ("nearly every day"). The scale is deemed a valid measure of depression severity and shows good agreement with diagnoses and other measures of depression (Martin et al., 2006). The official Dutch version of the PHQ-9 was given to Dutch participants.

*Subjective units of distress/discomfort (SUDS, cf. Wolpe & Lazarus, 1966).* The SUDS is a brief self-report instrument used to quickly and relatively unobtrusively determine a participant's subjective state of distress. Participants are required to rate their distress from 0 (no distress) to 100 (extreme distress). SUDS scales have shown convergent validity with other measures of distress/anxiety, and also proven sensitive to intervention effects (Foa et al., 1995; Kim et al., 2008; Soeter and Kindt, 2015; Tanner, 2012).

*Anxiety Sensitivity Index (ASI: Reiss, Peterson, Gursky, & McNally, 1986).* The ASI is a 16-item self-report scale measuring the degree to which a respondent fears feelings and behaviors associated with anxiety (i.e., anxiety sensitivity). Respondents rate how much they agree with statements reflecting anxiety sensitivity from 0 (very little) to 4 (very much). The robust psychometric properties of the ASI are reported in a review and the scale manual (Peterson and Plehn, 1999; Peterson and Reiss, 1992). Dutch participants were given a validated Dutch translation of the questionnaire with good internal consistency (*α* = .83: Vujanovic, Arrindell, Bernstein, Norton, & Zvolensky, 2007).

**Duration of exposure at treatment**

For the treatment sessions, we timed how long it was from when the door to the treatment room was opened, allowing the participant in, to when the door was closed after the participant completed the exposure. Independent groups ANOVAs indicated evidence against, or only equivocal evidence, for differences between groups in each experiment. In Experiment 1, there was a slight tendency for the scores in the enclosure condition to take slightly longer, perhaps because the procedure required slightly more explanation once the participant had entered the room. The Bayes Factors and *p* values in the model reflect the Bayes Factor favoring inclusion of ‘condition’ as an explanatory variable in a Bayesian ANOVA using default JASP priors, and the *p* value reflects the *p* value for a standard ANOVA comparing each condition.

Table S2. Timing of exposure in treatment session.

| Exp | Condition | Mean duration (mins) | SD duration (mins) | Median duration (mins) | Min (mins) | Max (mins) | *BF* condition | *p* condition |
| --- | --- | --- | --- | --- | --- | --- | --- | --- |
| 1 | standard | 3.94 | 2.17 | 3.94 | 1.87 | 7.75 | 1.04 | 0.09 |
| 1 | enclosure | 5.65 | 2.04 | 5.65 | 3.75 | 9.02 |  |  |
| 1 | loose tarantula | 3.45 | 1.30 | 3.45 | 1.95 | 6.67 |  |  |
| 1 | observe | 3.80 | 3.01 | 3.80 | 2.17 | 11.18 |  |  |
| 2 | placebo | 3.28 | 1.00 | 3.28 | 2.15 | 5.98 | 0.39 | 0.64 |
| 2 | propranolol | 3.11 | 0.76 | 3.11 | 2.23 | 4.88 |  |  |

**Assessment of confounds**

To assess the possibility of confounding influences from baseline measures that might affect the key regression models, we ran several checks, assessing both the relationships between SPQ, ASI, STAI-T, and PHQ z-scores with the starting scores of the variables involved in each regression, as well as with change scores in regression models that involved change over time. In all instances, p values and Bayes Factors for Kendall’s tau correlations indicated at most equivocal evidence for a relationship between variables. In the one instance where there was some evidence for a possible confound – ASI scores and BAT scores in Experiment 2, ASI scores were included in the regression model. For the longitudinal SPQ scores, differences were calculated from baseline relative to 4-, 8- and 12-weeks post-treatment in Experiment 1, and 4 weeks post-treatment in Experiment 2.

Table S3. Assessment of confounds.

| Variable pairing | Reference | tau | p value | Bayes Factor | n |
| --- | --- | --- | --- | --- | --- |
| s3.bat - asi.z | Exp 1, BATs | 0.137 | 0.284 | 0.445 | 43 |
| s3.bat - phq.z | Exp 1, BATs | -0.148 | 0.265 | 0.509 | 43 |
| s3.bat - stait.z | Exp 1, BATs | 0.019 | 0.889 | 0.201 | 43 |
| s3.bat - spq.z | Exp 1, BATs | 0.010 | 0.949 | 0.198 | 43 |
| distress.diff - asi.z | Exp 1, Distress | -0.093 | 0.405 | 0.289 | 43 |
| distress.diff - phq.z | Exp 1, Distress | 0.016 | 0.898 | 0.200 | 43 |
| distress.diff - stait.z | Exp 1, Distress | 0.068 | 0.541 | 0.242 | 43 |
| distress.diff - spq.z | Exp 1, Distress | -0.076 | 0.505 | 0.254 | 43 |
| s2.distress - asi.z | Exp 1, Distress | 0.033 | 0.787 | 0.207 | 43 |
| s2.distress - phq.z | Exp 1, Distress | 0.024 | 0.852 | 0.203 | 43 |
| s2.distress - stait.z | Exp 1, Distress | 0.041 | 0.729 | 0.213 | 43 |
| s2.distress - spq.z | Exp 1, Distress | 0.143 | 0.232 | 0.480 | 43 |
| **s3.bat - asi.z** | **Exp 2, BAT** | **-0.337** | **0.038** | **4.082** | **26** |
| s3.bat - phq.z | Exp 2, BAT | -0.137 | 0.426 | 0.399 | 26 |
| s3.bat - stait.z | Exp 2, BAT | -0.169 | 0.295 | 0.509 | 26 |
| s3.bat - spq.z | Exp 2, BAT | -0.127 | 0.447 | 0.374 | 26 |
| distress.diff - asi.z | Exp 2, Distress | -0.151 | 0.315 | 0.442 | 26 |
| distress.diff - phq.z | Exp 2, Distress | 0.104 | 0.510 | 0.329 | 26 |
| distress.diff - stait.z | Exp 2, Distress | -0.048 | 0.756 | 0.267 | 26 |
| distress.diff - spq.z | Exp 2, Distress | -0.110 | 0.474 | 0.339 | 26 |
| s2.distress - asi.z | Exp 2, Distress | 0.017 | 0.927 | 0.254 | 26 |
| s2.distress - phq.z | Exp 2, Distress | 0.069 | 0.677 | 0.284 | 26 |
| s2.distress - stait.z | Exp 2, Distress | -0.068 | 0.667 | 0.282 | 26 |
| s2.distress - spq.z | Exp 2, Distress | -0.108 | 0.493 | 0.336 | 26 |
| spq.4.diff - asi.z | Exp 1, SPQ | 0.066 | 0.575 | 0.246 | 39 |
| spq.6.diff - asi.z | Exp 1, SPQ | 0.154 | 0.226 | 0.488 | 34 |
| spq.8.diff - asi.z | Exp 1, SPQ | 0.109 | 0.399 | 0.330 | 33 |
| spq.10.diff - asi.z | Exp 1, SPQ | -0.034 | 0.815 | 0.243 | 30 |
| spq.12.diff - asi.z | Exp 1, SPQ | 0.160 | 0.221 | 0.507 | 32 |
| spq.4.diff - phq.z | Exp 1, SPQ | 0.136 | 0.265 | 0.426 | 39 |
| spq.6.diff - phq.z | Exp 1, SPQ | 0.028 | 0.843 | 0.227 | 34 |
| spq.8.diff - phq.z | Exp 1, SPQ | 0.040 | 0.775 | 0.237 | 33 |
| spq.10.diff - phq.z | Exp 1, SPQ | 0.035 | 0.813 | 0.244 | 30 |
| spq.12.diff - phq.z | Exp 1, SPQ | -0.005 | 0.987 | 0.228 | 32 |
| spq.4.diff - stait.z | Exp 1, SPQ | 0.161 | 0.166 | 0.568 | 39 |
| spq.6.diff - stait.z | Exp 1, SPQ | 0.030 | 0.823 | 0.228 | 34 |
| spq.8.diff - stait.z | Exp 1, SPQ | 0.084 | 0.513 | 0.283 | 33 |
| spq.10.diff - stait.z | Exp 1, SPQ | 0.150 | 0.266 | 0.453 | 30 |
| spq.12.diff - stait.z | Exp 1, SPQ | 0.052 | 0.696 | 0.248 | 32 |
| pre - asi.z | Exp 1, SPQ | 0.059 | 0.604 | 0.230 | 43 |
| pre - phq.z | Exp 1, SPQ | 0.031 | 0.796 | 0.206 | 43 |
| pre - stait.z | Exp 1, SPQ | -0.094 | 0.404 | 0.290 | 43 |
| spq.4.diff - asi.z | Exp 2, SPQ | 0.000 | 1.000 | 0.267 | 23 |
| spq.4.diff - phq.z | Exp 2, SPQ | 0.099 | 0.564 | 0.329 | 23 |
| spq.4.diff - stait.z | Exp 2, SPQ | -0.029 | 0.873 | 0.272 | 23 |
| pre - asi.z | Exp 2, SPQ | -0.063 | 0.686 | 0.278 | 26 |
| pre - phq.z | Exp 2, SPQ | 0.032 | 0.855 | 0.258 | 26 |
| pre - stait.z | Exp 2, SPQ | 0.154 | 0.304 | 0.451 | 26 |
| pre.6.diff - asi.z | Exp 1, Long-term SPQ | -0.011 | 0.960 | 0.263 | 24 |
| pre.14.diff - asi.z | Exp 1, Long-term SPQ | -0.159 | 0.345 | 0.451 | 21 |
| pre.6.diff - phq.z | Exp 1, Long-term SPQ | 0.055 | 0.743 | 0.280 | 24 |
| pre.14.diff - phq.z | Exp 1, Long-term SPQ | 0.159 | 0.356 | 0.451 | 21 |
| pre.6.diff - stait.z | Exp 1, Long-term SPQ | 0.261 | 0.085 | 1.202 | 24 |
| pre.14.diff - stait.z | Exp 1, Long-term SPQ | 0.124 | 0.465 | 0.374 | 21 |
| pre - asi.z | Exp 1, Long-term SPQ | 0.103 | 0.459 | 0.320 | 30 |
| pre - phq.z | Exp 1, Long-term SPQ | -0.021 | 0.898 | 0.238 | 30 |
| pre - stait.z | Exp 1, Long-term SPQ | -0.235 | 0.084 | 1.170 | 30 |
| pre.6.diff - asi.z | Exp 1, Long-term SPQ no exclusions | 0.027 | 0.851 | 0.237 | 31 |
| pre.14.diff - asi.z | Exp 1, Long-term SPQ no exclusions | -0.117 | 0.477 | 0.360 | 22 |
| pre.6.diff - phq.z | Exp 1, Long-term SPQ no exclusions | 0.075 | 0.591 | 0.274 | 31 |
| pre.14.diff - phq.z | Exp 1, Long-term SPQ no exclusions | 0.178 | 0.287 | 0.517 | 22 |
| pre.6.diff - stait.z | Exp 1, Long-term SPQ no exclusions | 0.104 | 0.433 | 0.320 | 31 |
| pre.14.diff - stait.z | Exp 1, Long-term SPQ no exclusions | 0.171 | 0.293 | 0.492 | 22 |
| pre - asi.z | Exp 1, Long-term SPQ no exclusions | 0.043 | 0.741 | 0.233 | 35 |
| pre - phq.z | Exp 1, Long-term SPQ no exclusions | 0.009 | 0.953 | 0.219 | 35 |
| pre - stait.z | Exp 1, Long-term SPQ no exclusions | -0.205 | 0.100 | 0.933 | 35 |

**Experiment 1 and 2:** **ANOVAs and contingency tables for baseline measures**

We used both Bayesian and frequentist ANOVAs to assess potential group differences in baseline measures of the STAIT, PHQ, ASI, SPQ, Age, and gender. As with other analyses, the Bayesian analyses used the default priors in *JASP*. All analyses simply included condition as a between subjects variable.

| Table S4. Bayesian ANOVA for STAIT in Experiment 1 | | | | | |  | Table S7. ANOVA for STAIT in Experiment 1 | | | | | |
| --- | --- | --- | --- | --- | --- | --- | --- | --- | --- | --- | --- | --- |
| Models | P(M) | P(M\|data) | BF _M_ | BF _10_ | error % |  | Cases | Sum of Squares | df | Mean Square | F | p |
| Null model | 0.5 | 0.634 | 1.735 | 1 |  |  | Condition | 278.371 | 3 | 92.79 | 1.787 | 0.166 |
| Condition | 0.5 | 0.366 | 0.577 | 0.58 | 7.938e -5 |  | Residuals | 2024.827 | 39 | 51.919 |  |  |
|  |  |  |  |  |  |  |  |  |  |  |  |  |
| Effects | P(incl) | P(incl\|data) | BF _incl_ |  |  |  |  |  |  |  |  |  |
| Condition | 0.5 | 0.366 | 0.577 |  |  |  |  |  |  |  |  |  |
|  |  |  |  |  |  |  |  |  |  |  |  |  |
| Table S5 Bayesian ANOVA for PHQ in Experiment 1 | | | | | |  | Table S8. ANOVA for PHQ in Experiment 1 | | | | | |
| Models | P(M) | P(M\|data) | BF _M_ | BF _10_ | error % |  | Cases | Sum of Squares | df | Mean Square | F | p |
| Null model | 0.5 | 0.769 | 3.336 | 1 |  |  | Condition | 25.014 | 3 | 8.338 | 1.031 | 0.389 |
| Condition | 0.5 | 0.231 | 0.3 | 0.3 | 0.004 |  | Residuals | 315.265 | 39 | 8.084 |  |  |
|  |  |  |  |  |  |  |  |  |  |  |  |  |
| Effects | P(incl) | P(incl\|data) | BF _incl_ |  |  |  |  |  |  |  |  |  |
| Condition | 0.5 | 0.231 | 0.3 |  |  |  |  |  |  |  |  |  |
|  |  |  |  |  |  |  |  |  |  |  |  |  |
| Table S6. Bayesian ANOVA for ASI in Experiment 1 | | | | | |  | Table S9. ANOVA for ASI in Experiment 1 | | | | | |
| Models | P(M) | P(M\|data) | BF _M_ | BF _10_ | error % |  | Cases | Sum of Squares | df | Mean Square | F | p |
| Condition | 0.5 | 0.656 | 1.906 | 1 |  |  | Condition | 416.711 | 3 | 138.904 | 3.047 | 0.04 |
| Null model | 0.5 | 0.344 | 0.525 | 0.53 | 1.340e -7 |  | Residuals | 1777.894 | 39 | 45.587 |  |  |
|  |  |  |  |  |  |  |  |  |  |  |  |  |
| Effects | P(incl) | P(incl\|data) | BF _incl_ |  |  |  |  |  |  |  |  |  |
| Condition | 0.5 | 0.656 | 1.906 |  |  |  |  |  |  |  |  |  |
|  |  |  |  |  |  |  |  |  |  |  |  |  |
| Table S10. Bayesian ANOVA for SPQ in Experiment 1 | | | | | |  | Table S13. ANOVA for SPQ in Experiment 1 | | | | | |
| Models | P(M) | P(M\|data) | BF _M_ | BF _10_ | error % |  | Cases | Sum of Squares | df | Mean Square | F | p |
| Null model | 0.5 | 0.732 | 2.737 | 1 |  |  | Condition | 36.025 | 3 | 12.008 | 1.241 | 0.308 |
| Condition | 0.5 | 0.268 | 0.365 | 0.37 | 1.928e -4 |  | Residuals | 377.417 | 39 | 9.677 |  |  |
|  |  |  |  |  |  |  |  |  |  |  |  |  |
| Effects | P(incl) | P(incl\|data) | BF _incl_ |  |  |  |  |  |  |  |  |  |
| Condition | 0.5 | 0.268 | 0.365 |  |  |  |  |  |  |  |  |  |
|  |  |  |  |  |  |  |  |  |  |  |  |  |
| Table S11. Bayesian ANOVA for Age in Experiment 1 | | | | | |  | Table S14. ANOVA for Age in Experiment 1 | | | | | |
| Models | P(M) | P(M\|data) | BF _M_ | BF _10_ | error % |  | Cases | Sum of Squares | df | Mean Square | F | p |
| Null model | 0.5 | 0.687 | 2.197 | 1 |  |  | Condition | 368.356 | 3 | 122.785 | 1.433 | 0.248 |
| Condition | 0.5 | 0.313 | 0.455 | 0.46 | 0.008 |  | Residuals | 3341.644 | 39 | 85.683 |  |  |
|  |  |  |  |  |  |  | *Note.*  Type III Sum of Squares | | | | | |
| Effects | P(incl) | P(incl\|data) | BF _incl_ |  |  |  |  |  |  |  |  |  |
| Condition | 0.5 | 0.313 | 0.455 |  |  |  |  |  |  |  |  |  |
|  |  |  |  |  |  |  | Table S15. Chi-square test for gender in Experiment 1 | | | | | |
| Table S12. Bayesian contingency table for gender in Experiment 1 | | | | | |  |  | Value | df | p |  |  |
|  |  |  | Value |  |  |  | Χ² | 1.831 | 3 | 0.608 |  |  |
| BF₁₀ Independent multinomial | | | 0.784 |  |  |  | N | 43 |  |  |  |  |
| N |  |  | 43 |  |  |  |  |  |  |  |  |  |

| Table S16. Bayesian ANOVA for STAIT in Experiment 2 | | | | | |  | Table S20. ANOVA for STAIT in Experiment 2 | | | | | |
| --- | --- | --- | --- | --- | --- | --- | --- | --- | --- | --- | --- | --- |
| Models | P(M) | P(M\|data) | BF _M_ | BF _10_ | error % |  | Cases | Sum of Squares | df | Mean Square | F | p |
| Null model | 0.5 | 0.704 | 2.384 | 1 |  |  | Condition | 20.346 | 1 | 20.346 | 0.39 | 0.54 |
| Condition | 0.5 | 0.296 | 0.42 | 0.42 | 2.317e -4 |  | Residuals | 1244.615 | 24 | 51.859 |  |  |
|  |  |  |  |  |  |  |  |  |  |  |  |  |
| Effects | P(incl) | P(incl\|data) | BF _incl_ |  |  |  |  |  |  |  |  |  |
| Condition | 0.5 | 0.296 | 0.42 |  |  |  |  |  |  |  |  |  |
|  |  |  |  |  |  |  |  |  |  |  |  |  |
| Table S17. Bayesian ANOVA for PHQ in Experiment 2 | | | | | |  | Table S21. ANOVA for PHQ in Experiment 2 | | | | | |
| Models | P(M) | P(M\|data) | BF _M_ | BF _10_ | error % |  | Cases | Sum of Squares | df | Mean Square | F | p |
| Null model | 0.5 | 0.611 | 1.572 | 1 |  |  | Condition | 3.846 | 1 | 3.846 | 1.54 | 0.23 |
| Condition | 0.5 | 0.389 | 0.636 | 0.64 | 0.004 |  | Residuals | 60 | 24 | 2.5 |  |  |
|  |  |  |  |  |  |  |  |  |  |  |  |  |
| Effects | P(incl) | P(incl\|data) | BF _incl_ |  |  |  |  |  |  |  |  |  |
| Condition | 0.5 | 0.389 | 0.636 |  |  |  |  |  |  |  |  |  |
|  |  |  |  |  |  |  |  |  |  |  |  |  |
| Table S18. Bayesian ANOVA for ASI in Experiment 2 | | | | | |  | Table S22. ANOVA for ASI in Experiment 2 | | | | | |
| Models | P(M) | P(M\|data) | BF _M_ | BF _10_ | error % |  | Cases | Sum of Squares | df | Mean Square | F | p |
| Null model | 0.5 | 0.633 | 1.726 | 1 |  |  | Condition | 36.962 | 1 | 36.962 | 1.28 | 0.27 |
| Condition | 0.5 | 0.367 | 0.58 | 0.58 | 0.002 |  | Residuals | 694 | 24 | 28.917 |  |  |
|  |  |  |  |  |  |  |  |  |  |  |  |  |
| Effects | P(incl) | P(incl\|data) | BF _incl_ |  |  |  |  |  |  |  |  |  |
| Condition | 0.5 | 0.367 | 0.58 |  |  |  |  |  |  |  |  |  |
|  |  |  |  |  |  |  |  |  |  |  |  |  |
| Table S19. Bayesian ANOVA for SPQ in Experiment 2 | | | | | |  | Table S23. ANOVA for SPQ in Experiment 2 | | | | | |
| Models | P(M) | P(M\|data) | BF _M_ | BF _10_ | error % |  | Cases | Sum of Squares | df | Mean Square | F | p |
| Null model | 0.5 | 0.674 | 2.063 | 1 |  |  | Condition | 9.846 | 1 | 9.846 | 0.79 | 0.38 |
| Condition | 0.5 | 0.326 | 0.485 | 0.49 | 1.817e -5 |  | Residuals | 300.615 | 24 | 12.526 |  |  |
|  |  |  |  |  |  |  |  |  |  |  |  |  |
| Effects | P(incl) | P(incl\|data) | BF _incl_ |  |  |  |  |  |  |  |  |  |
| Condition | 0.5 | 0.326 | 0.485 |  |  |  |  |  |  |  |  |  |
|  |  |  |  |  |  |  |  |  |  |  |  |  |
| Table S24. Bayesian ANOVA for Age in Experiment 2 | | | | | |  | Table S26. ANOVA for Age in Experiment 2 | | | | | |
| Models | P(M) | P(M\|data) | BF _M_ | BF _10_ | error % |  | Cases | Sum of Squares | df | Mean Square | F | p |
| Null model | 0.5 | 0.723 | 2.606 | 1 |  |  | Condition | 11.115 | 1 | 11.115 | 0.15 | 0.7 |
| Condition | 0.5 | 0.277 | 0.384 | 0.38 | 5.553e -5 |  | Residuals | 1760.923 | 24 | 73.372 |  |  |
|  |  |  |  |  |  |  | *Note.* Type III Sum of Squares | | | | | |
| Effects | P(incl) | P(incl\|data) | BF _incl_ |  |  |  |  |  |  |  |  |  |
| Condition | 0.5 | 0.277 | 0.384 |  |  |  |  |  |  |  |  |  |
|  |  |  |  |  |  |  |  |  |  |  |  |  |
| Table S25. Bayesian contingency table for gender in Experiment 2 | | | | | |  | Table S27. Chi-square test for gender in Experiment 2 | | | | | |
|  |  |  | Value |  |  |  |  | Value | df | p |  |  |
| BF₁₀ Independent multinomial | | | 0.944 |  |  |  | Χ² | 1.182 | 1 | 0.277 |  |  |
| N |  |  | 26 |  |  |  | N | 26 |  |  |  |  |
|  | | | |  |  |  |  |  |  |  |  |  |

**Experiments 1 and 2: ANOVAs for physiological measures**

For experiments 1 and 2, we ran Bayesian mixed measures ANOVAs with time (before receiving propranolol/’preprop’ vs. 90 minutes after receiving propranolol/‘postprop’) as a within subjects factor and condition (Experiment 2: Standard vs. Observe vs. Loose Tarantula vs. Enclosure, Experiment 2: Placebo vs. Propranolol) as within subjects factors. All ANOVAs used the default priors in *JASP*.

| Table S28. Bayesian Repeated Measures ANOVA for Systolic Blood Pressure in Experiment 1 | | | | | | |
| --- | --- | --- | --- | --- | --- | --- |
| Model Comparison |  |  |  |  |  |  |
| Models | P(M) | P(M\|data) | BF _M_ | BF _10_ | error % |  |
| Time | 0.2 | 0.591 | 5.782 | 1 |  |  |
| Time + Condition | 0.2 | 0.262 | 1.42 | 0.443 | 2.818 |  |
| Time + Condition + Time  ✻  Condition | 0.2 | 0.147 | 0.689 | 0.249 | 2.337 |  |
| Null model (incl. subject) | 0.2 | 1.713e -7 | 6.852e -7 | 2.898e -7 | 1.515 |  |
| Condition | 0.2 | 5.132e -8 | 2.053e -7 | 8.682e -8 | 1.58 |  |
| *Note.*  All models include subject |  |  |  |  |  |  |
|  |  |  |  |  |  |  |
| Analysis of Effects |  |  |  |  |  |  |
| Effects | P(incl) | P(incl\|data) | BF _incl_ |  |  |  |
| Time | 0.6 | 1 | 2.995e +6 |  |  |  |
| Condition | 0.6 | 0.409 | 0.461 |  |  |  |
| Time  ✻  Condition | 0.2 | 0.147 | 0.689 |  |  |  |
|  |  |  |  |  |  |  |
| Descriptives |  |  |  |  |  |  |
|  |  |  |  |  | 95% Credible Interval | |
| Time | Condition | Mean | SD | N | Lower | Upper |
| Postprop | standard | 122.875 | 13.214 | 12 | 114.48 | 131.27 |
|  | enclosure | 113.273 | 12.013 | 11 | 105.2 | 121.343 |
|  | loose | 120.083 | 11.564 | 12 | 112.74 | 127.431 |
|  | observe | 115.313 | 16.327 | 8 | 101.66 | 128.962 |
| Preprop | standard | 139.542 | 20.943 | 12 | 126.24 | 152.848 |
|  | enclosure | 133.636 | 12.002 | 11 | 125.57 | 141.7 |
|  | loose | 129.25 | 17.792 | 12 | 117.95 | 140.555 |
|  | observe | 127.75 | 18.398 | 8 | 112.37 | 143.131 |
|  |  |  |  |  |  |  |
| Table S29. Bayesian Repeated Measures ANOVA for Diastolic Blood Pressure in Experiment 1 | | | | | | |
| Model Comparison |  |  |  |  |  |  |
| Models | P(M) | P(M\|data) | BF _M_ | BF _10_ | error % |  |
| Time | 0.2 | 0.283 | 1.578 | 1 |  |  |
| Null model (incl. subject) | 0.2 | 0.24 | 1.265 | 0.849 | 0.809 |  |
| Time + Condition | 0.2 | 0.18 | 0.876 | 0.635 | 1.445 |  |
| Condition | 0.2 | 0.15 | 0.705 | 0.53 | 1.235 |  |
| Time + Condition + Time  ✻  Condition | 0.2 | 0.147 | 0.691 | 0.521 | 3.236 |  |
| *Note.*  All models include subject |  |  |  |  |  |  |
|  |  |  |  |  |  |  |
| Analysis of Effects |  |  |  |  |  |  |
| Effects | P(incl) | P(incl\|data) | BF _incl_ |  |  |  |
| Time | 0.6 | 0.61 | 1.042 |  |  |  |
| Condition | 0.6 | 0.477 | 0.608 |  |  |  |
| Time  ✻  Condition | 0.2 | 0.147 | 0.691 |  |  |  |
|  |  |  |  |  |  |  |
| Descriptives |  |  |  |  |  |  |
|  |  |  |  |  |  |  |
| Descriptives |  |  |  |  |  |  |
|  |  |  |  |  | 95% Credible Interval | |
| Time | Condition | Mean | SD | N | Lower | Upper |
| Postprop | standard | 78.625 | 10.785 | 12 | 71.772 | 85.478 |
|  | enclosure | 73.545 | 8.864 | 11 | 67.59 | 79.5 |
|  | loose | 80.958 | 10.914 | 12 | 74.024 | 87.893 |
|  | observe | 74.5 | 9.551 | 8 | 66.515 | 82.485 |
| Preprop | standard | 78.667 | 10.807 | 12 | 71.8 | 85.533 |
|  | enclosure | 81.727 | 9.498 | 11 | 75.346 | 88.108 |
|  | loose | 85.667 | 15.523 | 12 | 75.804 | 95.53 |
|  | observe | 73 | 8.928 | 8 | 65.536 | 80.464 |
|  |  |  |  |  |  |  |
| Table S30. Bayesian Repeated Measures ANOVA for Heart Rate in Experiment 1 | | | | | | |
| Model Comparison |  |  |  |  |  |  |
| Models | P(M) | P(M\|data) | BF _M_ | BF _10_ | error % |  |
| Time | 0.2 | 0.588 | 5.708 | 1 |  |  |
| Time + Condition | 0.2 | 0.331 | 1.976 | 0.562 | 2.839 |  |
| Time + Condition + Time  ✻  Condition | 0.2 | 0.081 | 0.354 | 0.138 | 2.168 |  |
| Null model (incl. subject) | 0.2 | 6.238e -12 | 2.495e -11 | 1.061e -11 | 0.804 |  |
| Condition | 0.2 | 1.708e -12 | 6.834e -12 | 2.906e -12 | 0.944 |  |
| *Note.*  All models include subject |  |  |  |  |  |  |
|  |  |  |  |  |  |  |
| Analysis of Effects |  |  |  |  |  |  |
| Effects | P(incl) | P(incl\|data) | BF _incl_ |  |  |  |
| Time | 0.6 | 1 | 8.391e +10 |  |  |  |
| Condition | 0.6 | 0.412 | 0.467 |  |  |  |
| Time  ✻  Condition | 0.2 | 0.081 | 0.354 |  |  |  |
|  |  |  |  |  |  |  |
| Descriptives |  |  |  |  |  |  |
|  |  |  |  |  | 95% Credible Interval | |
| Time | Condition | Mean | SD | N | Lower | Upper |
| Postprop | standard | 59.042 | 8.267 | 12 | 53.789 | 64.294 |
|  | enclosure | 58.318 | 4.285 | 11 | 55.439 | 61.197 |
|  | loose | 60.625 | 9.611 | 12 | 54.519 | 66.731 |
|  | observe | 53.188 | 6.579 | 8 | 47.687 | 58.688 |
| Preprop | standard | 79.292 | 17.075 | 12 | 68.443 | 90.141 |
|  | enclosure | 82.273 | 15.818 | 11 | 71.646 | 92.9 |
|  | loose | 80.833 | 21.704 | 12 | 67.043 | 94.623 |
|  | observe | 67.75 | 8.565 | 8 | 60.59 | 74.91 |

| Table S31. Bayesian Repeated Measures ANOVA for Systolic Blood Pressure in Experiment 2 | | | | | | |
| --- | --- | --- | --- | --- | --- | --- |
| Model Comparison | | | | | |  |
| Models | P(M) | P(M\|data) | BF _M_ | BF _10_ | error % |  |
| Time | 0.2 | 0.517 | 4.285 | 1 |  |  |
| Time + Condition | 0.2 | 0.292 | 1.651 | 0.565 | 2.518 |  |
| Time + Condition + Time  ✻  Condition | 0.2 | 0.191 | 0.942 | 0.368 | 2.867 |  |
| Null model (incl. subject) | 0.2 | 3.071e -5 | 1.229e -4 | 5.938e -5 | 1.119 |  |
| Condition | 0.2 | 1.341e -5 | 5.365e -5 | 2.593e -5 | 1.26 |  |
| *Note.*  All models include subject | | | | | |  |
|  |  |  |  |  |  |  |
| Analysis of Effects | | | |  |  |  |
| Effects | P(incl) | P(incl\|data) | BF _incl_ |  |  |  |
| Time | 0.6 | 1 | 15107.7 |  |  |  |
| Condition | 0.6 | 0.483 | 0.622 |  |  |  |
| Time  ✻  Condition | 0.2 | 0.191 | 0.942 |  |  |  |
|  |  |  |  |  |  |  |
| Descriptives | | | | | | |
|  | | | | | 95% Credible Interval | |
| Time | Condition | Mean | SD | N | Lower | Upper |
| Postprop | placebo | 118.962 | 11.596 | 13 | 111.95 | 125.969 |
|  | propranolol | 118.846 | 18.184 | 13 | 107.86 | 129.834 |
| Preprop | placebo | 129.615 | 14.835 | 13 | 120.65 | 138.58 |
|  | propranolol | 134.538 | 15.767 | 13 | 125.01 | 144.066 |
|  |  |  |  |  |  |  |
| Table S32. Bayesian Repeated Measures ANOVA for Diastolic Blood Pressure in Experiment 2 | | | | | | |
| Model Comparison | | | | | |  |
| Models | P(M) | P(M\|data) | BF _M_ | BF _10_ | error % |  |
| Time | 0.2 | 0.335 | 2.013 | 1 |  |  |
| Null model (incl. subject) | 0.2 | 0.234 | 1.222 | 0.699 | 0.972 |  |
| Time + Condition | 0.2 | 0.211 | 1.072 | 0.631 | 2.3 |  |
| Condition | 0.2 | 0.141 | 0.654 | 0.42 | 2.026 |  |
| Time + Condition + Time  ✻  Condition | 0.2 | 0.079 | 0.344 | 0.237 | 2.685 |  |
| *Note.*  All models include subject | | | | | |  |
|  |  |  |  |  |  |  |
| Analysis of Effects | | | |  |  |  |
| Effects | P(incl) | P(incl\|data) | BF _incl_ |  |  |  |
| Time | 0.6 | 0.625 | 1.113 |  |  |  |
| Condition | 0.6 | 0.431 | 0.505 |  |  |  |
| Time  ✻  Condition | 0.2 | 0.079 | 0.344 |  |  |  |
|  |  |  |  |  |  |  |
| Descriptives | | | | | | |
|  | | | | | 95% Credible Interval | |
| Time | Condition | Mean | SD | N | Lower | Upper |
| Postprop | placebo | 76.654 | 8.099 | 13 | 71.759 | 81.548 |
|  | propranolol | 79.538 | 12.796 | 13 | 71.806 | 87.271 |
| Preprop | placebo | 79.769 | 11.137 | 13 | 73.039 | 86.499 |
|  | propranolol | 82.231 | 14.043 | 13 | 73.745 | 90.717 |
|  |  |  |  |  |  |  |
| Table S33. Bayesian Repeated Measures ANOVA for Heart Rate in Experiment 2 | | | | | | |
| Model Comparison | | | | | |  |
| Models | P(M) | P(M\|data) | BF _M_ | BF _10_ | error % |  |
| Time | 0.2 | 0.408 | 2.757 | 1 |  |  |
| Time + Condition + Time  ✻  Condition | 0.2 | 0.407 | 2.74 | 0.996 | 2.837 |  |
| Time + Condition | 0.2 | 0.185 | 0.911 | 0.454 | 1.901 |  |
| Null model (incl. subject) | 0.2 | 2.586e -7 | 1.034e -6 | 6.338e -7 | 1.426 |  |
| Condition | 0.2 | 9.358e -8 | 3.743e -7 | 2.293e -7 | 1.923 |  |
| *Note.*  All models include subject | | | | | |  |
|  |  |  |  |  |  |  |
| Analysis of Effects | | | |  |  |  |
| Effects | P(incl) | P(incl\|data) | BF _incl_ |  |  |  |
| Time | 0.6 | 1 | 1.893e +6 |  |  |  |
| Condition | 0.6 | 0.592 | 0.967 |  |  |  |
| Time  ✻  Condition | 0.2 | 0.407 | 2.74 |  |  |  |
|  |  |  |  |  |  |  |
| Descriptives | | | | | | |
|  | | | | | 95% Credible Interval | |
| Time | Condition | Mean | SD | N | Lower | Upper |
| Postprop | placebo | 70.462 | 8.489 | 13 | 65.332 | 75.591 |
|  | propranolol | 62.808 | 10.752 | 13 | 56.31 | 69.305 |
| Preprop | placebo | 84.923 | 17.547 | 13 | 74.319 | 95.527 |
|  | propranolol | 87.846 | 13.957 | 13 | 79.412 | 96.28 |

**Regression model specification and priors**

Several Bayesian regression models were run to obtain the main results in the manuscript. Below we detail the model specification for each one, including the regression formulae and priors.

**Experiment 1 Behavioral Approach Test**

**Distribution modeled.** Cumulative ordinal with a probit link function.

**Regression formula.**

BAT score ~ Condition

**Priors.**

Table S34

| Parameter | Distribution | nu | Mean | SD |
| --- | --- | --- | --- | --- |
| Intercept | normal | - | 0 | 2 |
| Enclosure | normal | - | 0 | 1 |
| Loose T | normal | - | 0 | 1 |
| Observe | normal | - | 0 | 1 |

**Experiment 1 Distress scores**

**Distribution modeled.** Normal distribution with identity link (or, for the *t* model, a *t* distribution, with the same formula and priors)

**Regression formula.**

Distress ~ Condition + Session + Session*Condition + (1 | ppn)

**Priors.**

Table S35

| Parameter | Distribution | nu | Mean | SD |
| --- | --- | --- | --- | --- |
| Intercept | student t | 5 | 80 | 10 |
| Enclosure | student t | 5 | 0 | 12.5 |
| Loose T | student t | 5 | 0 | 12.5 |
| Observe | student t | 5 | 0 | 12.5 |
| Session | student t | 5 | 0 | 20 |
| Enclosure*Session | student t | 5 | 0 | 15 |
| Loose T*Session | student t | 5 | 0 | 15 |
| Observe*Session | student t | 5 | 0 | 15 |

**Experiment 1 SPQ scores in weekly follow-ups**

**Distribution modeled.** Normal distribution with identity link

**Regression formula.**

SPQ ~ Condition + Weeks since treatment + Weeks^2^ + Condition*Weeks + Condition*Weeks^2^ + (1 | ppn)

**Priors.**

Table S36

| Parameter | Distribution | nu | Mean | SD |
| --- | --- | --- | --- | --- |
| Intercept | normal | - | 22.5 | 6 |
| Enclosure | normal | - | 0 | 4 |
| Loose T | normal | - | 0 | 4 |
| Observe | normal | - | 0 | 4 |
| Weeks | normal | - | 0 | 3 |
| Weeks^2^ | normal | - | 0 | 0.5 |
| Enclosure*Weeks | normal | - | 0 | 3 |
| Loose T*Weeks | normal | - | 0 | 3 |
| Observe*Weeks | normal | - | 0 | 3 |
| Enclosure*Weeks^2^ | normal | - | 0 | 0.5 |
| Loose T*Weeks^2^ | normal | - | 0 | 0.5 |
| Observe*Weeks^2^ | normal | - | 0 | 0.5 |

**Experiment 1 SPQ scores in long-term follow-up**

**Distribution modeled.** Normal distribution with identity link

**Regression formula.**

SPQ ~ Time + Condition + Time*Condition + (1 | ppn)

**Priors.**

Table S37

| Parameter | Distribution | nu | Mean | SD |
| --- | --- | --- | --- | --- |
| Intercept | normal | - | 22.5 | 6 |
| Enclosure | normal | - | 0 | 4 |
| Loose T | normal | - | 0 | 4 |
| Observe | normal | - | 0 | 4 |
| 6m FU | normal | - | 0 | 3 |
| 14m FU | normal | - | 0 | 0.5 |
| Enclosure*6m FU | normal | - | 0 | 3 |
| Loose T*6m FU | normal | - | 0 | 3 |
| Observe*6m FU | normal | - | 0 | 3 |
| Enclosure*14m FU | normal | - | 0 | 0.5 |
| Loose T*14m FU | normal | - | 0 | 0.5 |
| Observe*14m FU | normal | - | 0 | 0.5 |

**Experiment 2 Behavioral Approach Test**

**Distribution modeled.** Cumulative ordinal with a probit link function.

**Regression formula.**

BAT score ~ Condition + ASI z-score

**Priors.**

Table S38

| Parameter | Distribution | nu | Mean | SD |
| --- | --- | --- | --- | --- |
| Intercept | normal | - | 0 | 2 |
| Propranolol | normal | - | 0 | 2 |
| ASI | normal | - | 0 | 2 |

**Experiment 2 Distress scores**

**Distribution modeled.** Normal distribution with identity link (or, for the *t* model, a *t* distribution, with the same formula and priors)

**Regression formula.**

Distress ~ Condition + Session + Session*Condition + (1 | ppn)

**Priors.**

Table S39

| Parameter | Distribution | nu | Mean | SD |
| --- | --- | --- | --- | --- |
| Intercept | student t | 5 | 80 | 10 |
| Propranolol | student t | 5 | 0 | 12.5 |
| Session | student t | 5 | 0 | 20 |
| Propranolol*Session | student t | 5 | 0 | 15 |

**Experiment 2 SPQ scores in weekly follow-ups**

**Distribution modeled.** Normal distribution with identity link

**Regression formula.**

SPQ ~ Condition + Weeks since treatment + Condition*Weeks + (1 | ppn)

**Priors.**

Table S40

| Parameter | Distribution | nu | Mean | SD |
| --- | --- | --- | --- | --- |
| Intercept | normal | - | 22.5 | 6 |
| Propranolol | normal | - | 0 | 4 |
| Weeks | normal | - | 0 | 3 |
| Propranolol *Weeks | normal | - | 0 | 3 |

**Long-term follow-ups in Experiment 1**

| Table S41. Regression parameter estimates for 6- and 14-month SPQ scores in Experiment 1 | | | | | | | |
| --- | --- | --- | --- | --- | --- | --- | --- |
| Parameter | Mean | SD | Lower 95% HDI | Upper 95% HDI | Rhat | Bulk ESS | Tail ESS |
| Intercept (Standard) | 22.36 | 1.73 | 18.94 | 25.67 | 1 | 13141 | 15670 |
| 6m FU | -8.47 | 2.01 | -12.29 | -4.4 | 1 | 13935 | 16488 |
| 14m FU | -9.58 | 2.08 | -13.7 | -5.53 | 1 | 15646 | 17885 |
| Enclosure | 0.53 | 2.51 | -4.39 | 5.51 | 1 | 15346 | 16379 |
| Loose tarantula | 0.74 | 2.42 | -3.96 | 5.59 | 1 | 13871 | 16502 |
| Observe | 0.24 | 2.36 | -4.46 | 4.73 | 1 | 14065 | 15571 |
| Enclosure, 6m FU | 0.6 | 3.09 | -5.32 | 6.79 | 1 | 18125 | 18166 |
| Enclosure, 14m FU | 4.41 | 3.91 | -3.22 | 12.12 | 1 | 22577 | 19641 |
| Loose tarantula, 6m FU | -4.95 | 3.06 | -11.04 | 1.02 | 1 | 17964 | 17554 |
| Loose tarantula, 14m FU | -3.85 | 3.1 | -9.9 | 2.25 | 1 | 17518 | 18093 |
| Observe, 6m FU | 2.35 | 2.83 | -3.27 | 7.76 | 1 | 15779 | 16529 |
| Observe, 14m FU | 1.85 | 3.47 | -5.17 | 8.52 | 1 | 19208 | 18257 |
|  |  |  |  |  |  |  |  |
| SD of ppn intercept | 4.24 | 1.01 | 2.31 | 6.29 | 1 | 6296 | 7374 |
|  |  |  |  |  |  |  |  |
| sigma | 4.28 | 0.55 | 3.26 | 5.38 | 1 | 8274 | 10752 |
| SD = standard deviation, HDI = Highest Density Interval, ESS = Effective sample size. | | | | | | | |

**
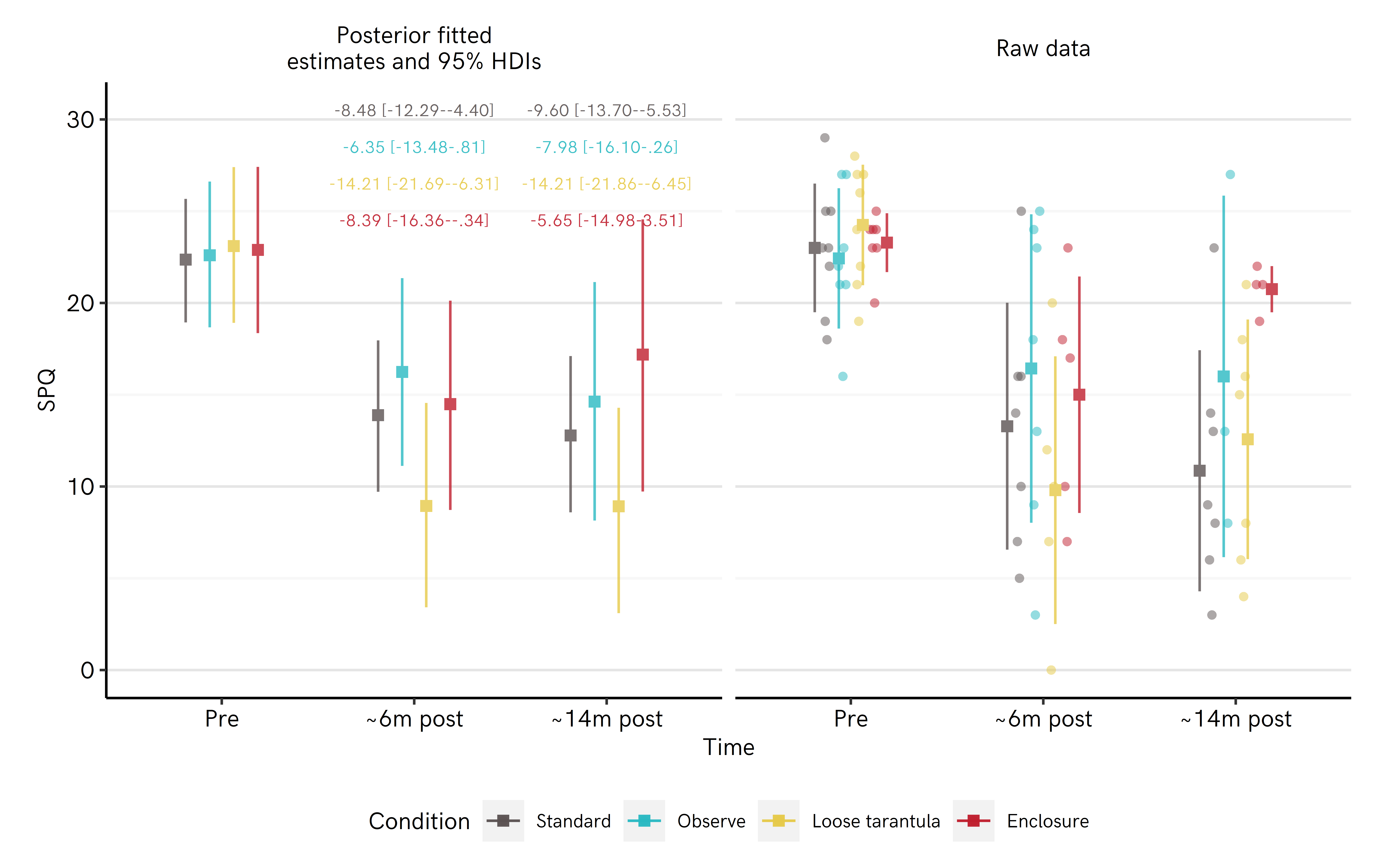
**

Figure S1. Change in SPQ scores in each condition at long-term follow-up, in Experiment 1. Left panel shows mean posterior parameter estimates from a Bayesian regression model (square points) and their 95% Highest Density Intervals (HDI: whiskers), and mean difference scores [95% HDIs] vs. pretreatment as text. Right panel shows the raw data points (round points), with the mean (square points) +/- 1 standard deviation (whiskers). Pre = Pre-treatment, post = post-treatment.

| Table S42. Regression parameter estimates for 6- and 14-month SPQ scores in Experiment 1, with no exclusions | | | | | | | |
| --- | --- | --- | --- | --- | --- | --- | --- |
| Parameter | Mean | SD | Lower 95% HDI | Upper 95% HDI | Rhat | Bulk ESS | Tail ESS |
| Intercept | 23.6 | 1.49 | 20.73 | 26.56 | 1 | 11212 | 14949 |
| 6m FU | -10.75 | 1.86 | -14.22 | -6.96 | 1 | 12520 | 16666 |
| 14m FU | -12.13 | 1.95 | -15.95 | -8.31 | 1 | 14258 | 16164 |
| Enclosure | 0.12 | 2.1 | -3.93 | 4.26 | 1 | 13081 | 16149 |
| Loose tarantula | 0.7 | 2.11 | -3.37 | 4.79 | 1 | 12674 | 15560 |
| Observe | -0.63 | 2.22 | -4.9 | 3.78 | 1 | 13976 | 15910 |
| Enclosure, 6m FU | 1.94 | 2.74 | -3.6 | 7.09 | 1 | 15307 | 17822 |
| Enclosure, 14m FU | 7.71 | 3.23 | 1.38 | 14.08 | 1 | 17854 | 18355 |
| Loose tarantula, 6m FU | -2.03 | 2.8 | -7.45 | 3.54 | 1 | 15907 | 17962 |
| Loose tarantula, 14m FU | 0.35 | 2.86 | -5.06 | 6.09 | 1 | 16806 | 17822 |
| Observe, 6m FU | 4.25 | 2.85 | -1.49 | 9.72 | 1 | 15709 | 17765 |
| Observe, 14m FU | 4.03 | 3.52 | -2.87 | 10.86 | 1 | 18252 | 18041 |
|  |  |  |  |  |  |  |  |
| SD of ppn intercept | 3.63 | 0.87 | 2.02 | 5.48 | 1 | 6643 | 7956 |
|  |  |  |  |  |  |  |  |
| sigma | 4.57 | 0.5 | 3.69 | 5.6 | 1 | 8791 | 12123 |
| SD = standard deviation, HDI = Highest Density Interval, ESS = Effective sample size. | | | | | | | |

**Long-term follow-ups in Experiment 2**

Independent samples t-tests were conducted for comparisons of SPQ and FSQ scores at 2- and 6-months post-treatment in Experiment 2. Below, we present analyses including all participants, or restricted to those who responded within 30 days of the 2-month follow-up, or 45 days of the 6-month follow-up. The modified SPQ scale was used, and is analyzed below either as a scale – utilizing the Likert response format – or are a binary measure, with any form of agreement/disagreement being rated as True/False. Bayesian analyses used the default priors in JASP.

| Table S43. Long-term follow-ups for Experiment 2, with exclusions for date of response | | | | | | | |
| --- | --- | --- | --- | --- | --- | --- | --- |
|  | BF₁₀ | error % | t | df | p |  |  |
| 2 months SPQ scored binary | 0.395 | 0.025 | -0.163 | 19 | 0.87 |  |  |
| 2 months SPQ with Likert | 0.405 | 0.025 | -0.312 | 19 | 0.76 |  |  |
| 2 months FSQ | 1.043 | 0.004 | -1.693 | 19 | 0.11 |  |  |
| 6 months SPQ scored binary | 0.423 | 2.976e -6 | -0.155 | 15 | 0.88 |  |  |
| 6 months SPQ with Likert | 0.422 | 1.442e -6 | -0.108 | 15 | 0.92 |  |  |
| 6 months FSQ | 0.609 | 0.003 | -1.068 | 16 | 0.3 |  |  |
|  |  |  |  |  |  |  |  |
|  |  |  |  |  |  | 95% Credible Interval | |
|  | Group | N* | Mean | SD | SE | Lower | Upper |
| 2 months SPQ scored binary | placebo | 11 | 15.09 | 7.368 | 2.22 | 10.141 | 20.041 |
|  | propranolol | 10 | 15.6 | 6.851 | 2.17 | 10.699 | 20.501 |
| 2 months SPQ with Likert | placebo | 11 | -2.682 | 26.78 | 8.07 | -20.673 | 15.309 |
|  | propranolol | 10 | 1.3 | 31.62 | 10 | -21.321 | 23.921 |
| 2 months FSQ | placebo | 11 | 51.36 | 19.71 | 5.94 | 38.123 | 64.604 |
|  | propranolol | 10 | 68.9 | 27.47 | 8.69 | 49.253 | 88.547 |
| 6 months SPQ scored binary | placebo | 8 | 18.5 | 5.831 | 2.06 | 13.625 | 23.375 |
|  | propranolol | 9 | 19 | 7.297 | 2.43 | 13.391 | 24.609 |
| 6 months SPQ with Likert | placebo | 8 | 10.5 | 24.48 | 8.66 | -9.968 | 30.968 |
|  | propranolol | 9 | 11.94 | 29.99 | 10 | -11.11 | 34.998 |
| 6 months FSQ | placebo | 8 | 61.38 | 14.93 | 5.28 | 48.895 | 73.855 |
|  | propranolol | 10 | 72 | 24.67 | 7.8 | 54.355 | 89.645 |
| * responses were excluded if they did not take place within 30 days of the 2m follow-up, or 45 days of the 6m follow-up | | | | | | | |
|  |  |  |  |  |  |  |  |
| Table S44. Long-term follow-ups for Experiment 2, with no exclusions | | | | | | | |
|  | BF₁₀ | error % | t | df | p |  |  |
| 2 months SPQ scored binary | 0.388 | 1.497e -4 | -0.216 | 21 | 0.83 |  |  |
| 2 months SPQ with Likert | 0.395 | 1.662e -4 | -0.309 | 21 | 0.76 |  |  |
| 6 months SPQ scored binary | 0.423 | 2.976e -6 | -0.155 | 15 | 0.88 |  |  |
| 6 months SPQ with Likert | 0.43 | 3.310e -5 | -0.352 | 16 | 0.73 |  |  |
| 2 months FSQ | 0.968 | 0.002 | -1.634 | 21 | 0.12 |  |  |
| 6 months FSQ | 0.609 | 0.003 | -1.068 | 16 | 0.3 |  |  |
|  |  |  |  |  |  |  |  |
|  |  |  |  |  |  | 95% Credible Interval | |
|  | Group | N* | Mean | SD | SE | Lower | Upper |
| 2 months SPQ scored binary | placebo | 13 | 14.92 | 7.879 | 2.19 | 10.162 | 19.684 |
|  | propranolol | 10 | 15.6 | 6.851 | 2.17 | 10.699 | 20.501 |
| 2 months SPQ with Likert | placebo | 13 | -2.654 | 29.4 | 8.15 | -20.42 | 15.112 |
|  | propranolol | 10 | 1.3 | 31.62 | 10 | -21.321 | 23.921 |
| 6 months SPQ scored binary | placebo | 8 | 18.5 | 5.831 | 2.06 | 13.625 | 23.375 |
|  | propranolol | 9 | 19 | 7.297 | 2.43 | 13.391 | 24.609 |
| 6 months SPQ with Likert | placebo | 9 | 7.389 | 24.73 | 8.24 | -11.621 | 26.399 |
|  | propranolol | 9 | 11.94 | 29.99 | 10 | -11.11 | 34.998 |
| 2 months FSQ | placebo | 13 | 51.85 | 22.63 | 6.28 | 38.171 | 65.522 |
|  | propranolol | 10 | 68.9 | 27.47 | 8.69 | 49.253 | 88.547 |
| 6 months FSQ | placebo | 8 | 61.38 | 14.93 | 5.28 | 48.895 | 73.855 |
|  | propranolol | 10 | 72 | 24.67 | 7.8 | 54.355 | 89.645 |
| * no restrictions were made on inclusion of responses | | | | | | | |

**Additional regression model output**

| Table S45. Regression parameter estimates for Distress scores using a *t* distribution in Experiment 1 | | | | | | | |
| --- | --- | --- | --- | --- | --- | --- | --- |
| Parameter | Mean | SD | Lower 95% HDI | Upper 95% HDI | Rhat | Bulk ESS | Tail ESS |
| Intercept | 89.29 | 3.66 | 81.79 | 96.18 | 1 | 21533 | 20078 |
| Enclosure | 0.64 | 5.16 | -9.63 | 10.72 | 1 | 25238 | 19157 |
| Loose tarantula | -0.66 | 5.04 | -10.53 | 9.2 | 1 | 24016 | 18846 |
| Observe | -3.13 | 5.63 | -14.49 | 7.64 | 1 | 26307 | 19747 |
| Post-treatment | -24.3 | 5.05 | -34.33 | -14.46 | 1 | 19645 | 18470 |
| Enclosure, Post-treatment | -8.22 | 7.25 | -22.38 | 5.89 | 1 | 23286 | 19799 |
| Loose tarantula, Post-treatment | -11.55 | 7.19 | -25.25 | 3.09 | 1 | 22909 | 18998 |
| Observe, Post-treatment | 5.08 | 7.42 | -9.24 | 19.92 | 1 | 26869 | 18882 |
|  |  |  |  |  |  |  |  |
| SD of ppn intercept | 5.45 | 2.89 | 0 | 10.12 | 1 | 5111 | 9365 |
|  |  |  |  |  |  |  |  |
| sigma | 13.36 | 2.04 | 9.12 | 17.21 | 1 | 9537 | 11626 |
| nu | 13.43 | 11.08 | 1.43 | 35.87 | 1 | 19276 | 15834 |
| SD = standard deviation, HDI = Highest Density Interval, ESS = Effective sample size. | | | | | | | |

| Table S46. Regression parameter estimates for Distress scores with the tarantula at post-treatment Experiment 1 | | | | | | | |
| --- | --- | --- | --- | --- | --- | --- | --- |
| Parameter | Mean | SD | Lower 95% HDI | Upper 95% HDI | Rhat | Bulk ESS | Tail ESS |
| Intercept | 88.48 | 3.8 | 81.04 | 95.97 | 1 | 17838 | 18692 |
| Enclosure | 2.17 | 5.43 | -8.07 | 13.23 | 1 | 19513 | 18136 |
| Loose tarantula | -0.16 | 5.34 | -10.62 | 10.28 | 1 | 18902 | 18580 |
| Observe | -2.74 | 5.87 | -14.41 | 8.53 | 1 | 20808 | 18649 |
| Post-treatment | -23.58 | 4.85 | -33.34 | -14.31 | 1 | 15329 | 18768 |
| Enclosure, Post-treatment | 3.66 | 6.87 | -9.84 | 17.1 | 1 | 19331 | 18716 |
| Loose tarantula, Post-treatment | -14.68 | 6.95 | -28.74 | -1.41 | 1 | 18947 | 18291 |
| Observe, Post-treatment | 4.65 | 7.44 | -10.17 | 19.18 | 1 | 19736 | 18287 |
|  |  |  |  |  |  |  |  |
| SD of ppn intercept | 6.23 | 3.01 | 0.06 | 11 | 1 | 3966 | 7093 |
|  |  |  |  |  |  |  |  |
| sigma | 14.65 | 1.55 | 11.7 | 17.66 | 1 | 6976 | 14022 |
| SD = standard deviation, HDI = Highest Density Interval, ESS = Effective sample size. | | | | | | | |

| Table S47. Regression parameter estimates for weekly SPQ scores in Experiment 1, linear model | | | | | | | |
| --- | --- | --- | --- | --- | --- | --- | --- |
| Parameter | Mean | SD | Lower 95% HDI | Upper 95% HDI | Rhat | Bulk ESS | Tail ESS |
| Intercept | 19.95 | 1.49 | 17.02 | 22.92 | 1 | 3092 | 5410 |
| Enclosure | -1.31 | 2.15 | -5.44 | 2.95 | 1 | 3530 | 6244 |
| Loose tarantula | -1.5 | 2.05 | -5.48 | 2.6 | 1 | 3457 | 6496 |
| Observe | 0.07 | 2.25 | -4.35 | 4.52 | 1 | 3772 | 7008 |
| Weeks | -0.54 | 0.07 | -0.67 | -0.41 | 1 | 7128 | 11167 |
| Enclosure, Weeks | 0.19 | 0.1 | 0.01 | 0.38 | 1 | 9211 | 13970 |
| Loose tarantula, Weeks | 0.18 | 0.09 | 0 | 0.35 | 1 | 8711 | 12461 |
| Observe, Weeks | 0.35 | 0.1 | 0.15 | 0.55 | 1 | 8705 | 13623 |
|  |  |  |  |  |  |  |  |
| SD of ppn intercept | 6.09 | 0.71 | 4.89 | 7.66 | 1 | 3084 | 5450 |
|  |  |  |  |  |  |  |  |
| sigma | 3.39 | 0.11 | 3.19 | 3.62 | 1 | 13900 | 15939 |
| SD = standard deviation, HDI = Highest Density Interval, ESS = Effective sample size. | | | | | | | |

| Table S48. Regression parameter estimates for weekly SPQ scores in Experiment 2, quadratic model | | | | | | | |
| --- | --- | --- | --- | --- | --- | --- | --- |
| Parameter | Mean | SD | Lower 95% HDI | Upper 95% HDI | Rhat | Bulk ESS | Tail ESS |
| Intercept | 23.52 | 1.31 | 20.95 | 26.1 | 1 | 6092 | 10672 |
| Propranolol | -1.11 | 1.76 | -4.51 | 2.42 | 1 | 6580 | 11007 |
| Weeks | -0.99 | 0.61 | -2.19 | 0.2 | 1 | 8700 | 13923 |
| Weeks2 | -0.12 | 0.1 | -0.31 | 0.07 | 1 | 8535 | 13244 |
| Propranolol, Weeks | -0.92 | 0.87 | -2.63 | 0.8 | 1 | 8423 | 12669 |
| Propranolol, Weeks2 | 0.26 | 0.15 | -0.02 | 0.56 | 1 | 8695 | 13161 |
|  |  |  |  |  |  |  |  |
| SD of ppn intercept | 3.95 | 0.69 | 2.72 | 5.34 | 1 | 5763 | 10320 |
|  |  |  |  |  |  |  |  |
| sigma | 3.6 | 0.24 | 3.16 | 4.08 | 1 | 17694 | 17802 |
| SD = standard deviation, HDI = Highest Density Interval, ESS = Effective sample size. | | | | | | | |

**Trust in treatment**

Although we did not use a standardized/validated measure, we did request a single 8-point Likert scale response regarding how much participants trusted in the experimental procedure, at the end of the intake session. Table S49 shows the descriptive statistics for each group, and Table S50 shows correlations between the trust score and various outcomes measures. It can be seen that this measure of trust does not reliably correlate with any baseline scores or change in scores over time. It should be noted that this measure was taken before direct contact with the clinician in the treatment session, who generally provided further explanation and justification of the procedure, and may have increased the participants’ confidence in the treatment.

Table S49. Reported trust in the experimental treatment across groups.

| Condition | mean | sd | median |
| --- | --- | --- | --- |
| Standard | 5.58 | 1.16 | 6 |
| Enclosure | 5.91 | 1.51 | 6 |
| Loose | 6 | 1.04 | 6 |
| Observe | 6.25 | 0.886 | 6.5 |
|  |  |  |  |
| Placebo | 6.23 | 1.59 | 7 |
| Propranolol | 5.54 | 1.27 | 5 |

Table S50. Correlations between various outcome measures and trust scores.

| Variable pairing | Reference | tau | p value | n |
| --- | --- | --- | --- | --- |
| Trust - s3.BAT | Exp 1, BATs | 0.00178 | 1 | 43 |
| Trust – baseline distress | Exp 1, Distress | 0.0168 | 0.902 | 43 |
| Trust - distress change | Exp 1, Distress | -0.0103 | 0.939 | 43 |
| Trust - 4 week SPQ change | Exp 1, SPQ | -0.00314 | 0.99 | 39 |
| Trust - 6 week SPQ change | Exp 1, SPQ | 0.111 | 0.42 | 34 |
| Trust - 8 week SPQ change | Exp 1, SPQ | 0.134 | 0.336 | 33 |
| Trust - 10 week SPQ change | Exp 1, SPQ | -0.0825 | 0.577 | 30 |
| Trust - 12 week SPQ change | Exp 1, SPQ | -0.0345 | 0.814 | 32 |
| Trust - Baseline SPQ | Exp 1, SPQ | -0.14 | 0.247 | 43 |
| Trust - s3.BAT | Exp 2, BATs | 0.258 | 0.132 | 26 |
| Trust – baseline distress | Exp 2, Distress | 0.0447 | 0.797 | 26 |
| Trust - distress change | Exp 2, Distress | -0.17 | 0.281 | 26 |
| Trust - 4 week SPQ change | Exp 2, SPQ | -0.112 | 0.51 | 23 |
| Trust - Baseline SPQ | Exp 2, SPQ | -0.216 | 0.174 | 26 |
| SPQ = spider phobia questionnaire, s3 BAT = post-treatment behavioral approach test | | | | |
